# Identification of a Potential miRNA–mRNA Regulatory Network Associated With the Prognosis of HBV-ACLF

**DOI:** 10.3389/fmolb.2021.657631

**Published:** 2021-04-28

**Authors:** Shanshan Ma, Zhongyang Xie, Lingjian Zhang, Ya Yang, He Jiang, Xiaoxi Ouyang, Yalei Zhao, Qiuhong Liu, Xiaowei Xu, Lanjuan Li

**Affiliations:** ^1^State Key Laboratory for Diagnosis and Treatment of Infectious Diseases, The First Affiliated Hospital, College of Medicine, Zhejiang University, Hangzhou, China; ^2^National Clinical Research Center for Infectious Diseases, The First Affiliated Hospital, College of Medicine, Zhejiang University, Hangzhou, China; ^3^Collaborative Innovation Center for Diagnosis and Treatment of Infectious Diseases, The First Affiliated Hospital, College of Medicine, Zhejiang University, Hangzhou, China; ^4^Department of Infectious Diseases, The First Affiliated Hospital, College of Medicine, Zhejiang University, Hangzhou, China

**Keywords:** hepatitis B virus, acute-on-chronic liver failure, prognosis, network, miRNA

## Abstract

**Background:**

Hepatitis B virus-related acute-on-chronic liver failure (HBV-ACLF) is a life-threatening disease with a high mortality rate; the systemic inflammatory response plays a vital role in disease progression. We aimed to determine if a miRNA–mRNA co-regulatory network exists in the peripheral blood mononuclear cells (PBMCs) of HBV-ACLF patients, which might be important for prognosis.

**Methods:**

Transcriptome-wide microRNA (miRNA) and mRNA microarrays were used to define the miRNA and mRNA expression profiles of the PBMCs of HBV-ACLF patients in a discovery cohort. The targets of the miRNAs were predicted. We built a miRNA-mRNA regulatory network through bioinformatics analysis, and used quantitative real-time polymerase chain reaction (qRT-PCR) to assess the importance of candidate miRNAs and mRNAs. We also assessed the direct and transcriptional regulatory effects of miRNAs on target mRNAs using a dual-luciferase reporter assay.

**Results:**

The miRNA/mRNA PBMC expression profiles of the discovery cohort, of whom eight survived and eight died, revealed a prognostic interactive network involving 38 miRNAs and 313 mRNAs; this was constructed by identifying the target genes of the miRNAs. We validated the expression data in another cohort, of whom 43 survived and 35 died; miR-6840-3p, miR-6861-3p, JADE2, and NR3C2 were of particular interest. The levels of miR-6840-3p and miR-6861-3p were significantly increased in the PBMCs of the patients who died, and thus predicted prognosis (areas under the curve values = 0.665 and 0.700, respectively). The dual-luciferase reporter assay indicated that miR-6840-3p directly targeted JADE2.

**Conclusion:**

We identified a prognostic miRNA-mRNA co-regulatory network in the PBMCs of HBV-ACLF patients. miR-6840-3p-JADE2 is a potential miRNA–mRNA pair contributing to a poor prognosis.

## Introduction

Acute-on-chronic liver failure (ACLF) is a life-threatening disease characterized by acute deterioration of liver function in patients with pre-existing chronic liver disease, and hepatic or extrahepatic organ failure (Bernal et al., 2015). The short-term mortality rate is 40–60% (Seto et al., 2018). The incidence rates of ACLF and acute liver failure vary worldwide. In China and most other Asian countries, hepatitis B virus (HBV) is the most common cause of ACLF; chronic HBV infection is prevalent in Asia (Sarin et al., 2014). Few effective treatments for liver failure are available. Thus, it is essential to discover biomarkers that can identify HBV-ACLF patients at high risk of poor prognosis. In terms of ACLF pathogenesis, the systemic inflammation (SI) hypothesis proposes that ACLF is attributable to exacerbation of SI and the associated systemic circulatory dysfunction, which triggers organ failure because of hypoperfusion and the deleterious effects of inflammatory mediators on organ microcirculation and homeostasis (Bernardi et al., 2015; Clària et al., 2016). Peripheral blood mononuclear cells (PBMCs), which are important components of the immune system, play pivotal roles in inflammation and immune system deterioration. A previous study described the differential transcriptome profiles of the PBMCs of healthy controls, and patients with chronic hepatitis B infections and HBV-ACLF (Zhou et al., 2016), but did not explore the relationship between transcriptional status and the prognosis of HBV-ACLF.

MicroRNAs (miRNAs) are small, endogenous, single-stranded non-coding RNAs approximately 19–25 nucleotides in length that function as key post-transcriptional regulators in a variety of cellular processes, including differentiation, proliferation, migration, apoptosis, stress, and the immune response (Giovannetti et al., 2012; Wu et al., 2018). Recent studies indicated that miRNAs might play a role in the pathogenesis of liver injury, and showed that miR-197 in PBMCs may re-activate liver inflammation (Chen et al., 2013). In addition, it’s reported that two regulatory pathways, miR-93-5p-JUN and miR-106b-5p-STAT3 were identified to contribute to the tumorigenesis of HBV-related hepatocellular carcinoma based on the potential miRNA–mRNA regulatory network (Lou et al., 2019). However, the changes in miRNA levels and possible regulation of mRNA expression in HBV-ACLF patients remain to be elucidated. To the best of our knowledge, no systemic and comprehensive analysis of miRNA–mRNA co-regulation has been conducted in the context of the prognosis of HBV-ACLF patients. Thus, no useful markers of HBV-ACLF progression or prognosis are available.

Combination of microarray technologies and bioinformatic analysis can provide researchers with unprecedent convenience in seeking novel biomarkers and therapeutic targets. We speculate that as a result of immune disorder, the miRNA–mRNA of PBMC in HBV-ACLF patients may be abnormal, either. In this study, we explored the miRNA–mRNA regulatory networks of PBMCs in HBV-ACLF patients with different prognoses. We defined the inflammatory changes that affected prognosis and aimed to identify informative prognostic biomarkers for early recognition of disease progression and severity. New biomarkers could aid disease management and interventions to reduce the high mortality rate.

## Materials and Methods

### Study Population

All of the enrolled HBV-ACLF patients were admitted to the First Affiliated Hospital of the Medical School of Zhejiang University between April 2017 and October 2019. HBV-ACLF was diagnosed using the criteria of the Chinese Group on the Study of Severe Hepatitis B-ACLF (COSSH-ACLF) (Wu et al., 2018). Sixteen subjects, of whom eight survived and eight died, underwent PBMC miRNA and mRNA microarray profiling. Also, 43 survival controls and 35 death cases were recruited for validation. All patients were grouped according to whether or not they survives for 28 days without liver transplantation.

The exclusion criteria were as follows: (1) drug-induced or autoimmune hepatitis, alcoholic liver disease, or an acute fatty liver caused by pregnancy or a hemolytic condition; (2) a congenital metabolic liver disease, such as Wilson’s disease or hemochromatosis; (3) super-infection or co-infection with hepatitis A, C, D, or E viruses or human immunodeficiency virus; and, (4) other fatal diseases, including malignancy.

### Clinical and Follow-Up Data

Basic clinical information was collected from all patients at the time of first admission. Age, sex, height, weight, and blood pressure were recorded, as well as blood test results. Routine laboratory measurements included blood cell count, conventional liver biochemical evaluation, coagulopathy status, kidney function assessment, measurement of sodium (Na) and alpha-fetoprotein (AFP) levels, and HBV serological testing. Clinical outcomes at 28 and 90 days were recorded, and patients were evaluated using various prognostic scoring systems including the Chronic Liver Failure-Consortium-ACLF score (CLIF-C ACLF), the COSSH-ACLF, the Model for End-Stage Liver Disease (MELD), and the MELD-Na.

### PBMC and RNA Isolation

Peripheral blood mononuclear cells were isolated using the Ficoll-Histopaque technique (Sigma Aldrich, St. Louis, MO, United States) within 4 h of blood collection. Total RNA of PBMCs was extracted using TRIzol reagent (Invitrogen, Carlsbad, CA, United States) following the manufacturer’s instructions, and then dissolved in DNase/RNase-free water and stored at –80°C.

### Transcriptome-Wide miRNA and mRNA Expression Profiling

Transcriptome-wide miRNA and mRNA expression profiles were determined using the Agilent Human miRNA Microarray Kit, release 21.0, 8 × 60 K (Design ID: 070156; Agilent Technologies, San Diego, CA, United States) and the Agilent SurePrint G3 Human Gene Expression ver. 3 8 × 60K Microarray (Design ID: 072363), respectively. Total RNA was quantified on a NanoDrop ND-2000 device (Thermo Scientific, Waltham, MA, United States) and RNA integrity was assessed using an Agilent Bioanalyzer 2100 (Agilent). Sample labeling, microarray hybridization, and washing were performed as recommended by the manufacturer. Briefly, total RNA was transcribed into double-stranded cDNA, and cRNA labeled with cyanine-3-CTP was synthesized and hybridized to the microarray. After washing, the arrays were scanned using the Scanner G2505C Microarray Scanner System (Agilent Technologies).

### Transcriptome-Wide miRNA and mRNA Expression Profiling

Agilent Feature Extraction software (ver. 10.7.1.1; Agilent Technologies) was used to obtain raw data. GeneSpring (ver. 14.8; Agilent Technologies) was then employed for basic analysis. Raw data were initially normalized using the quantile algorithm. Probes with at least one (of a possible two) “Detected” flags were further analyzed. Differentially expressed miRNAs (DEMIRs) and differentially expressed genes (DEGs) were identified by calculating fold changes (FCs) and *P*-values using the *t*-test. The thresholds for up- and down-regulated miRNAs and mRNAs were a FC ≥ 2.0 and *P*-value ≤ 0.05.

### Bioinformatics Analysis

We then used the Gene Ontology (GO) and Kyoto Encyclopedia of Genes and Genomes (KEGG) databases to determine the roles played by differentially expressed mRNAs. Fisher exact test was used to calculate the enrichment significance of each term and KEGG enrichment analysis used hypergeometric distribution to calculate the association degree of each pathway in keggpath with this differential gene. miRDB and miRWalk were used to predict genes targeted by the identified miRNAs (Sticht et al., 2018; Chen and Wang, 2020). We combined the mRNA microarray data with information on these genes. More narrowly, only predicted target genes of up-regulated DE-miRNAs were significantly down-regulated in results of DEGs while predicted targets genes of down-regulated DE-miRNAs were significantly up-regulated in results of DEGs were identified as potential target genes. With these data, we built a potential miRNA-mRNA regulatory network, and visualized it using Cytoscape software^1^. And Pearson’s correlation analysis between miRNA and mRNA was conducted.

### Validation

For validation, the expression levels of miRNAs and mRNAs of interest, in the PBMCs of HBV-ACLF patients in the validation cohort, were determined via quantitative real-time polymerase chain reaction (qRT-PCR). We first selected 13 significant DEMIRs with a *P*-value < 0.01, FC ≥ 5.0, or match-mRNA value ≥ 20. We combined the data on these miRNA-targeted genes with the mRNA microarray data. The total RNA of the validation cohort was reverse-transcribed into cDNA using the Prime Script RT Master Mix (TaKaRa, Japan) and the Mir-X miRNA First-Strand Synthesis kit (TaKaRa, Japan). miRNA and mRNA qPCR-specific primers (Sangon, Shanghai, China) were then synthesized. Next, qPCR was performed in triplicate using an QuantStudio 3 Real-Time PCR system (Thermo Scientific) and TB Green Premix Ex Taq II (TaKaRa, Japan). The expression levels of miRNAs (relative to U6) and mRNAs (relative to β-actin) were analyzed using the 2^–*ddCt*^ method.

### Cell Transfection and Dual-Luciferase Reporter Assay

Human embryonic kidney cells (293T line; American Type Culture Collection, Manassas, VA, United States) exhibit high-level transfection efficiency and were thus used to perform the dual-luciferase reporter assay. The 293T cells were cultured in Dulbecco’s modified Eagle’s medium (DMEM) supplemented with 10% (v/v) fetal bovine serum (FBS) at 37°C in a humidified atmosphere under 5% (v/v) CO_2_. Prior to transfection, 293T cells were seeded into 96-well platshuanes at 1 × 10^4^ cells/well and grown for 24 h. Then, the pmiR-RB-Report vector (RiboBio, Guangzhou, China) with the 3′-untranslated region (UTR) of the wild-type or mutant target gene was co-transfected with miRNA mimics or the negative control into the cells. Dual-luciferase activity was measured 48 h later using the Dual-Luciferase Reporter Assay System (Promega, Madison, WI, United States). The activity of firefly luciferase was normalized to that of Renilla luciferase to control for differences in transfection efficiency among experiments. The dual-luciferase reporter plasmids, miRNA mimics, and mimic negative control were all from RiboBio Co. Ltd.

### Statistical Analysis

All statistical tests were performed using SPSS software (ver. 26.0; SPSS Inc., Chicago, IL, United States). Summary statistics for continuous variables with non-normal distributions are presented as medians (25th and 75th percentiles); normally distributed variables are presented as means ± standard deviations (SDs). Student’s *t*-test, the non-parametric Mann–Whitney *U* test, or Kruskal–Wallis test was performed as appropriate to compare continuous variables. Categorical variables are presented as numbers (with percentages) and were compared using the χ^2^ test or Fisher’s exact test. Receiver operating characteristic (ROC) curve analysis was used to determine whether differences between specific miRNA levels affected the survival of HBV-ACLF patients, based on the area under the curve (AUC) values. A *P*-value < 0.05 was considered to indicate significance.

## Results

### Analysis of Prognosis-Related Differentially Expressed miRNAs and mRNAs

**FIGURE 1:**
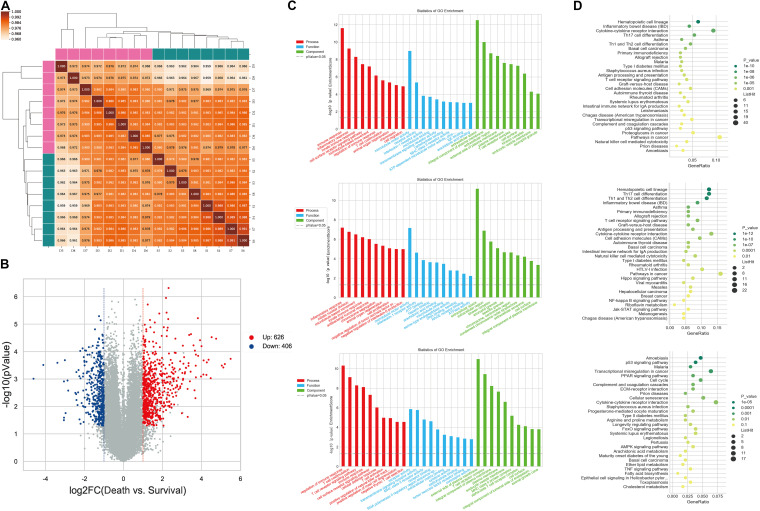
Differential expression of PBMC mRNAs between death group and survival group in the discovery cohort. **(A)** The correlation coefficient graph shows the similarity of the expression patterns of the samples in the group. **(B)** The Volcano plot of DEGs. The red spots indicate significantly upregulated mRNAs, and the blue spots indicate significantly downregulated mRNAs. **(C)** Gene ontology analysis of DEGs. The higher the bar chart height, the smaller the corresponding *P*-value. Red indicates biological process, blue indicates molecular function, and green indicates cellular component. **(D)** Pathways analysis of DEGs. The horizontal axis shows the enrichment degree and the vertical axis represents the enrichment pathway. The larger spots indicates that the more genes fall into the pathway and the greener the color, the higher the significance of enrichment.

The expression profiles of miRNAs and mRNAs in PBMCs from 16 HBV-ACLF patients with different 28-day outcomes (eight who survived and eight who died) were analyzed. The clinical characteristics of this cohort are provided in Supplementary Table 1. For mRNA expression profiling, the mRNA data from all 16 samples were analyzed using Pearson correlation; all coefficients were significant (Figure 1A). At the thresholds *P* ≤ 0.05 and FC ≥ 2, 1032 mRNAs were determined to exhibit difference in expression between survival group and death group (Supplementary Table 2). In total, 626 and 406 mRNAs were up- and down-regulated, respectively, in patients who died compared to those who lived. A volcano plot revealed the variance in DEGs (Figure 1B). GO analysis showed that the DEGs were enriched in certain shared biological functions. In the biological processes (BPs) category, the enriched terms included the immune, inflammatory, and innate immune responses. We analyzed the relevant molecular activities via KEGG pathway analysis of the DEGs. The enriched pathways were cytokine-cytokine receptor interaction, Th17 cell differentiation, and immune system-related diseases including inflammatory bowel disease (IBD) and asthma. The GO terms and DEG pathways are shown in Figures 1C,D.

**FIGURE 2:**
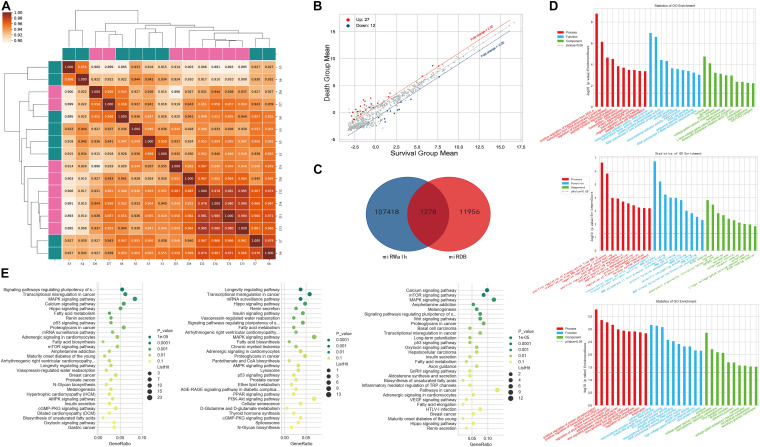
Differential expression of PBMC miRNAs between death and survival groups in the discovery cohort. **(A)** The correlation coefficient graph shows the similarity of the expression patterns of the samples in the group. **(B)** The scatter plot of DEMIRs. The red spots indicate significantly upregulated miRNAs, and the blue spots indicate significantly downregulated miRNAs. **(C)** The intersection of the predicted target genes of DEMIRs from two databases. **(D)** The gene ontology annotation for the predicted target genes of DEMIRs. The higher the bar chart height, the smaller the corresponding *P*-value. Red indicates biological process, blue indicates molecular function, and green indicates cellular component. **(E)** Pathways enrichment analysis for the predicted target genes of DEMIRs. The horizontal axis shows the enrichment degree and the vertical axis represents the enrichment pathway. The larger spots indicates that the more predicted target genes fall into the pathway and the greener the color, the higher the significance of enrichment.

The miRNA correlation results are shown in Figure 2A. Of the 2,598 miRNAs detected, 39 were significantly differentially expressed between the two groups; 27 were upregulated and 12 were downregulated in patients who died compared to those who survived (Figure 2B). We used miRDB and miRWalk to predict the target mRNAs of these 39 miRNAs and obtained 1,278 miRNA/mRNA pairs (Figure 2C). GO analysis showed that the predicted mRNAs were enriched in terms of the regulation and activation of the RNA polymerase II promoter and other BPs. KEGG pathway enrichment analysis revealed that the target mRNAs were enriched in cancer pathways, stem cells, and MAPK signaling. The GO terms and pathways of the target mRNAs of DEMIRs are shown in Figures 2D,E, respectively.

### Integrative Analysis of DEGs and DEMIRs, and Construction of a Prognosis-Related Interaction Network

**FIGURE 3:**
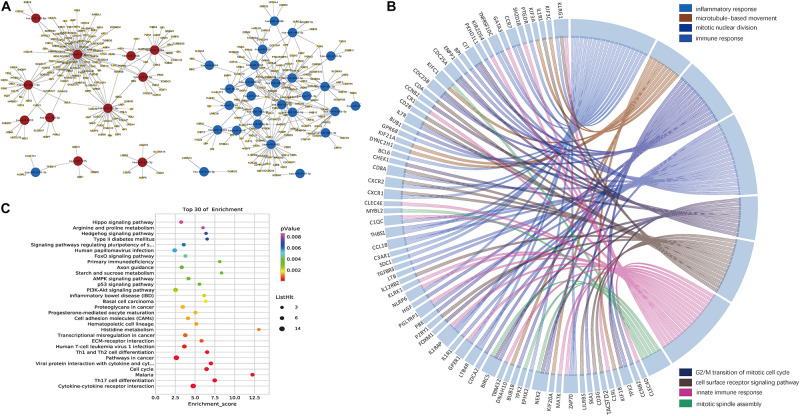
Networks constructed by the DEMIRs and DEGs. **(A)** Regulatory networks constructed by all negatively correlated miRNAs and target mRNAs (listed in Supplementary Table 3). Combining DEGs and predicted target genes of DEMIRs data, 38 miRNAs exhibiting both differential expression and negative regulation of target genes were selected. **(B)** Gene ontology chord plot of top eight significant gene ontology terms belonging to the biological process for the miRNA–mRNA regulatory network associated with the prognosis of HBV-ACLF. The genes are linked to their assigned terms via colored ribbons. **(C)** Pathways enrichment analysis for genes. The vertical axis indicates the enriched pathways, and the horizontal axis indicates the number of differential genes in a specific pathway in that pathway.

We then identified the PBMC miRNA–mRNA regulatory pathways involved in HBV-ACLF progression. It is known that an inverse relationship exists between miRNA and target mRNA expression (Giray et al., 2014). Thus, we combined data on the 1,278 significant miRNA–mRNA pairs predicted by the two databases with those on the detected 1,032 DEGs. The primary network constructed by Cytoscape software included 38 miRNAs and their 313 target mRNAs (Figure 3A and Supplementary Tables 3,4).

The top eight BPs included the immune system, the response to inflammation, and mitosis, which had many genes in common. IL1RAP, CXCR1, CXCR2, and IL7R were all associated with immunity and the response to inflammation. NEK2 and BIRC5 are involved in several processes, including mitotic nuclear division, the G2/M transition of the mitotic cell cycle, and mitotic spindle assembly (Figure 3B). The top five KEGG signaling pathways associated with a poor prognosis included cytokine-cytokine receptor interaction, Th17 cell differentiation, malaria, the cell cycle, and viral protein interaction with cytokines and cytokine receptors (Figure 3C). Several of the KEGG functional categories pertained to the immune system.

### Validation of the DEMIRs and DEGs

**FIGURE 4:**
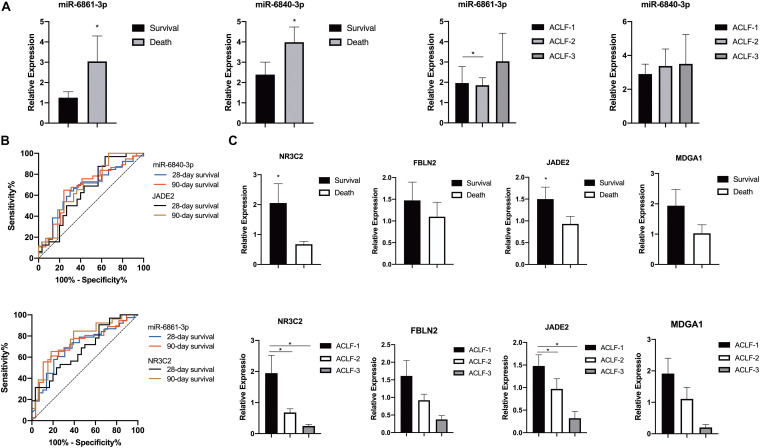
Differential expressions of miRNAs in the validation cohort. **(A)** Expression of miR-6861-3p and miR-6840-3p in PBMCs of patients with HBV-ACLF in different prognoses or severe grades. **(B)** ROC curve analyses of miR-6861-3p, miR-6840-3p, NR3C2 and JADE2 expressions of patients with HBV-ACLF in different prognoses. **(C)** Expression of NR3C2 and JADE2 in PBMCs of patients with HBV-ACLF in different prognoses or severe grades. *P < 0.05.

The miRNA-mRNA regulatory network was identified bioinformatically. To validate the network, the expression levels of the DEMIRs and DEGs in PBMCs of 78 patients with HBV-ACLF, of whom 35 died and 43 survived, were assayed. The clinical characteristics of the validation group are provided in Table 1. We first selected 13 significant DEMIRs with a *P*-value < 0.01, FC ≥ 5.0, or match-mRNA value ≥ 20 (Table 2). qRT-PCR showed that miR-6861-3p (*P* = 0.003) and miR-6840-3p (*P* = 0.02) were significantly differentially expressed between the patients who survived and those died (Figure 4A), consistent with the above data. The miRNAs that were not significantly differentially expressed, and those with changes in expression opposite to those of the discovery cohort, are shown in Supplementary Figures 1, 2.

**TABLE 1:** Clinical characteristics of patients with HBV-ACLF in validation cohort.

**Characteristics**	**Survival group (*N* = 43)**	**Death group (*N* = 35)**	***P*-value**
Age (years)	44 ± 11	50 ± 13	0.025
Male (no.)	88.4% (38)	85.7% (30)	0.746
BMI (kg/m^2^)	24.0 ± 2.7	24.3 ± 4.0	0.777
**HBV-DNA level (IU/ml)**			
<200	2.3% (1)	8.6% (3)	0.109
200−2 × 10^4^	27.9% (12)	11.4% (4)	
2 × 10^4^−2 × 10^6^	39.6% (17)	31.4% (11)	
≥ 2 × 10^6^	30.2% (13)	48.6% (17)	
**Laboratory data**			
WBC (10^9^/L)	7.8 ± 3.0	8.9 ± 3.1	0.110
Hb (g/L)	125.0 ± 18.7	123.9 ± 18.3	0.778
PLT (10^9^/L)	100 (73, 132)	98 (70, 133)	0.651
ALB (g/L)	30.3 (28.8, 32.4)	29.9 (28.0, 34.8)	0.960
ALT (U/L)	200 (100, 360)	258 (108, 398)	0.388
AST (U/L)	116 (68, 165)	149 (90, 367)	0.047
TB (μmol/L)	361.2 (286.1, 398.5)	398.4 (292.5, 517.3)	0.093
Cr (μmol/L)	65 (53, 75)	77 (59, 140)	0.008
Na (mmol/L)	137 (135, 139)	137 (133, 139)	0.559
INR	1.85 (1.64, 2.18)	2.70 (2.00, 3.40)	< 0.001
AFP (μg/L)	148.3 (30.1, 393.7)	73.6 (26.9, 182.2)	0.105
**Severity score**			
COSSH-ACLFs	5.9 (5.5, 6.4)	6.7 (6.3, 7.7)	< 0.001
CLIF-C ACLFs	40.7 ± 8.1	41.4 ± 6.2	0.660
MELD	21.6 (19.9, 23.9)	29.1 (23.7, 34.5)	< 0.001
MELD-Na	21.3 (17.8, 23.9)	29.1 (21.7, 35.6)	< 0.001

*Data are mean ± SD, median (p25, p75), or number (percentage). Comparisons were performed by *t-*test, Mann-Whitney *U*-test, χ^2^ test or Fisher’s exact test.BMI, body mass index; HBV, hepatitis B virus; ACLF, acute-on-chronic liver failure; WBC, white blood cell; Hb, hemoglobin; PLT, platelet; ALB, albumin; ALT, alanine transferase; AST, aspartate aminotransferase; TB, total bilirubin; Cr, creatinine; INR, international normalized ratio; AFP, alpha fetoprotein; COSSH, the Chinese Group on the Study of Severe Hepatitis B; MELD, model for end-stage liver disease; Na, sodium.*

**TABLE 2:** The selected differentially expressed miRNAs and mRNAs between death group versus survival group in HBV-ACLF patients for validation by qRT-PCR.

	**Regulation**	**log_2_^*FC*^**	***P*-value**	**Number of target mRNA**
**miRNA**				
hsa-miR-6840-3p	Up	3.002	0.019	20
hsa-miR-6861-3p	Up	2.369	0.004	4
hsa-miR-6085	Up	1.127	< 0.001	28
hsa-miR-664a-3p	Up	2.936	< 0.001	10
hsa-miR-5010-3p	Up	2.798	0.001	1
hsa-miR-4436b-5p	Up	2.773	0.001	13
hsa-miR-1268a	Down	–2.445	0.004	17
hsa-miR-3656	Down	–2.906	0.004	6
hsa-miR-4739	Down	–2.193	0.006	94
hsa-miR-4484	Up	2.733	0.007	4
hsa-miR-642a-3p	Up	3.273	0.010	9
hsa-miR-29b-1-5p	Down	–1.320	0.017	20
hsa-miR-361-3p	Down	–1.513	0.048	30
**mRNA**				
JADE2	Down	–1.350	< 0.001	
MDGA1	Down	–2.968	0.017	
FBLN2	Down	–1.676	< 0.001	
NR3C2	Down	–1.161	< 0.001	

*HBV, hepatitis B virus; ACLF, acute-on-chronic liver failure; qRT-PCR, quantitative real time polymerase chain reaction.*

Receiver operating characteristic curve analysis was used to assess the potential utility of miR-6861-3p and miR-6840-3p as biomarkers predicting the 28- and 90-day transplant-free survival rates of HBV-ACLF patients. As shown in Figure 4B, the AUC values for distinguishing between 28- and 90-day death and survival were 0.700 (95% confidence interval [CI]: 0.573–0.827, *P* = 0.005) and 0.729 (95% CI: 0.603–0.855, *P* = 0.002) for miR-6861-3p, and 0.665 (95% CI: 0.535–0.795, *P* = 0.020) and 0.680 (95% CI: 0.548–0.813, *P* = 0.012) for miR-6840-3p, respectively.

The expression levels of four potential targets (based on their *P*-values and FCs; Table 2) were determined (Figure 4C). Compared to the survival group, the NR3C2 (*P* = 0.016) and JADE2 (*P* = 0.043) expression levels were significantly lower in patients who died; although not statistically significant, the FBLN2 (*P* = 0.204) and MDGA1 (*P* = 0.089) expression levels were also lower in these patients. Besides, the AUC values for distinguishing between 28- and 90-day death and survival were 0.650 (95% CI: 0.511–0.790, *P* = 0.042) and 0.685 (95% CI: 0.550–0.820, *P* = 0.015) for JADE2, and 0.678 (95% CI: 0.546–0.810, *P* = 0.016) and 0.765 (95% CI: 0.642–0.887, *P* < 0.001) for NR3C2, respectively.

### miR-6840-3p Directly Targets JADE2

**FIGURE 5:**
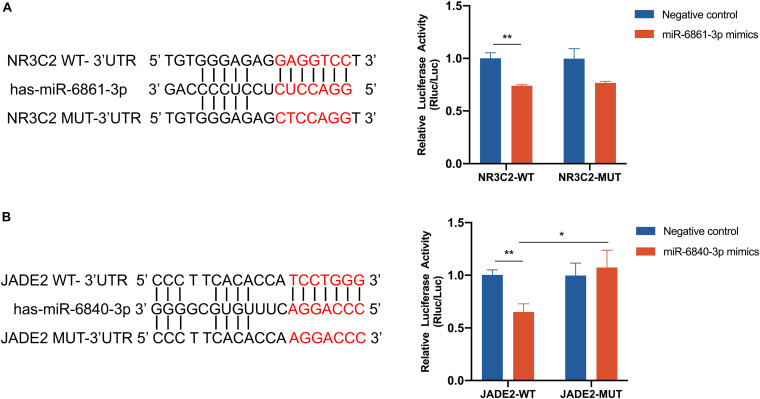
Dual luciferase assay of 293T cells co-transfected with the pmiR-RB-Report vector containing the wild-type or mutant-type target gene 3′ UTR and miRNA mimics or negative control. **(A)** NR3C2 and miR-6861-3p. **(B)** JADE2 and miR-6840-3p. *P < 0.05, **P < 0.01.

NR3C2 and JADE2 mRNAs are putative molecular targets of miR-6861-3p and miR-6840-3p, respectively. The ability of these miRNAs to regulate the 3′-UTRs of these genes was evaluated using the dual-luciferase reporter assay. Direct binding of the miRNA to the pmiR-RB-Report vector with the target gene 3′-UTR represses luciferase activity in 293T cells (Zhang et al., 2019; Garcia-Lacarte et al., 2019). Cells were co-transfected with luciferase reporter plasmids containing wild-type or mutant target gene 3′-UTRs and miRNA mimics or negative controls. Compared to the negative control, overexpression of miR-6861-3p significantly inhibited the luciferase activity of the pmiR-RB-Report vector with the wild-type NR3C2 3′-UTR (*P* = 0.001), but not that of the vector with the mutant NR3C2 3′-UTR (*P* = 0.074) (Figure 5A). In addition, the miR-6840-3p mimic decreased the luciferase activity of the pmiR-RB-Report vector with the wild-type JADE2 3′-UTR by 35% (*P* = 0.002), but not that of the vector with the mutant JADE2 3′-UTR (*P* = 0.015) (Figure 5B). Thus, JADE2 was shown to be a downstream target of miR-6840-3p.

## Discussion

A previous study suggested that SI played an important role in ACLF, and that SI severity was highly correlated with ACLF severity and 28- and 90-day mortality rates (Clària et al., 2016). PBMCs are peripheral immune cells, and include lymphocytes and monocytes. We observed inflammatory and immune system changes in the PBMCs of HBV-ACLF patients. HBV infection modulates the expression of host miRNAs, thus further regulating the mRNAs involved in the development of HBV-related liver diseases (Deng and Lu, 2016). Therefore, we studied the actions of miRNAs in the PBMCs of HBV-ACLF patients.

Recently, miRNA–mRNA regulatory networks have been shown to be involved in the onset and progression of several human diseases, including HBV-related hepatocellular carcinoma and schizophrenia. These networks facilitate exploration of the underlying pathogenesis, which may lead to effective therapies (Lou et al., 2019; Santarelli et al., 2019). In schizophrenia, a significant miRNA–mRNA interaction network was identified, including miR-92a, miR-495, and miR-134, which converged with differentially expressed genes in pathways involved in neurodevelopment and oligodendrocyte function (Santarelli et al., 2019). However, the miRNA–mRNA co-regulatory network involved in HBV-ACLF prognosis remained unknown. We first analyzed the PBMC miRNAs and mRNAs of HBV-ACLF patients, and then performed bioinformatic analyses and experimental validation. A regulatory network with 38 miRNAs and 313 mRNAs was established to understand the molecular mechanism of disease progression and facilitate the prediction of prognosis. The abnormalities identified affected immunity, the response to inflammation, and mitosis. Pathway analysis emphasized the importance of cytokine-cytokine receptor interaction. Immune system abnormalities played an important role in HBV-ACLF progression.

We found that miR-6840-3p-JADE2 was associated with a poor HBV-ALCF prognosis. Also, miR-6861-3p and NR3C2 were up- and down-regulated, respectively, in those patients who died compared to those who lived. Xu et al. (2019) showed that miR-6840-3p bound to ZEB2-AS1 and then targeted PLXNB1 to promote the progression of laryngeal squamous cell carcinoma. JADE2 (gene for apoptosis and differentiation in epithelia 2) is a member of the small JADE family (Panchenko, 2016). Several studies found that JADE2 was associated with histone modification in ovarian cancer and acquired immune deficiency syndrome (Quintela et al., 2019; Maxwell et al., 2020). JADE-2 also acted as an E3 ubiquitin ligase of lysine-specific demethylase-1 (LSD1) in neural cells; a glucocorticoid induced JADE2-mediated LSD1 degradation (Han et al., 2014; Anan et al., 2018). However, the function of JADE2 in HBV-ACLF remains unknown. We found that mir-6840-3p and JADE2 levels were significantly increased and decreased, respectively, in both patients who died and patients with ACLF-3 grade disease. Moreover, the direct relationship between miR-6840-3p and JADE2 was verified by the dual-luciferase reporter assay. How dysregulation of miR-6840-3p-JADE2 ins PBMCs leads to disease progression remains unclear. *Ex vivo* studies with freshly isolated PBMCs found that the expression of proinflammatory cytokines in response to lipopolysaccharide (LPS) was higher in cells from patients with cirrhosis than in those of healthy subjects (Gandoura et al., 2013). We speculate that this may be a factor in ACLF pathogenesis, as cytokines are involved in inflammation. ROC curve analysis revealed the utility of miR-6840-3p as a novel biomarker predicting the 28- and 90-day liver transplant-free survival rates of HBV-ACLF patients.

miR-6861-3p was an early biomarker of prognosis in HBV-ACLF patients. Intense research on miRNA has been conducted in recent years and the role of miRNAs in ACLF has been discussed. Tao et al. reported that increased circulating miR-125b-5p was associated with the severity of liver damage; a high serum miR-125b-5p level may predict poor outcomes in HBV-ACLF patients (Tao et al., 2019). Reduced serum miR-223-3p and miR-25-3p levels were associated with ACLF and poor prognosis (Cisilotto et al., 2020). Several studies (including ours) have assessed the miRNA expression profile of ACLF, but the results have been inconsistent (Ding et al., 2015; Wen et al., 2018). One study used high-throughput sequencing to construct a comprehensive PBMC miRNA profile of patients with HBV-ACLF; qRT-PCR validation confirmed that the expression levels of six miRNAs (miR-21-5p, miR-34c-5p, miR-143-3p, miR-143-5p, miR-374a-3p, and miR-542-3) were elevated compared to those of patients with chronic hepatitis B (CHB) and healthy controls (Ding et al., 2015). Another report concluded that the expression levels of miR-146a-5p, miR-122-3p, and miR-328-3p were positively correlated with the severity of liver inflammation in patients with ACLF, and may be useful to predict HBV-ACLF severity (Wen et al., 2018). Several explanations of the inconsistency results are plausible. First, the patient selection criteria differed among studies. Our study is the first to use the COSSH criteria to select HBV-ACLF patients. Second, both serum and PBMC samples have been studied. Finally, the detection method also differed among (high-throughput sequencing vs. microarrays).

NR3C2 (nuclear receptor subfamily 3 group C member 2), also known as mineralocorticoid receptor (MR), is a cytoplasmic steroid-responsive receptor that controls electrolyte and water balance (Horisberger and Rossier, 1992). In recent years, many studies have found that NR3C2 serves as a tumor suppressor in many cancers, including hepatocellular carcinoma (Nie et al., 2015; Yang et al., 2019). Although NR3C2 status in ACLF has not been explored, MR mRNA and protein expression were reduced in the hepatocytes of patients with cirrhosis and ascites (Schreier et al., 2018). In HBV-ACLF patients, we found that NR3C2 expression was significantly lower among both those with a poor prognosis and those with ACLF-3 grade disease. NR3C2 activation, translocation, and degradation in HBV-ACLF patients requires further study.

Although we performed a comprehensive analysis and experimental validation of the miRNA-mRNA co-regulatory network, in terms of its effect on the prognosis of HBV-ACLF patients, and successfully identified several potential miRNA–mRNA pathways that may also affect the prognosis, our study had the following limitations: (1) The sample sizes of the microarray and validation cohorts were not large. (2) The analysis of the relationships among miRNAs, targeted mRNAs, and related pathways require more further experimental confirmation both *in vitro* and *in vivo*. (3) Transcriptome-wide miRNA and mRNA expression profiles were used to describe the network between miRNAs and possible target mRNAs and their overall biological functions, in which the role of protein was not involved. From this point, it’s a pity that experimental techniques such as crosslinking-immunprecipitation and high-throughput sequencing (CLIP-seq) have not been applied, which could identify the interaction network of RNAs and RNA binding proteins (RBPs) in the whole genome and verify the direct interactions between miRNAs and target proteins and the precise binding sites.

## Conclusion

We identified a miRNA–mRNA co-regulatory network in PBMCs from HBV-ACLF patients with prognostic value. miR-6840-3p-JADE2 may promote ACLF progression and thus a poor prognosis, and miR-6840-3p and miR-6861-3p may also have prognostic value. We hope that our findings will facilitate future in-depth studies and improve our understanding of the molecular mechanisms of HBV-ACLF.

## Data Availability Statement

The raw data of microarrays for miRNA and mRNA expression profiles have been submitted to the GEO database as required with accession numbers GSE168046 and GSE168048.

## Ethics Statement

The studies involving human participants were reviewed and approved by the Ethics Committee of the First Affiliated Hospital of the Medical School of Zhejiang University. The patients/participants provided their written informed consent to participate in this study.

## Author Contributions

SM, ZX, XX, and LL had the idea for and designed the study and take responsibility for the integrity of the data and the accuracy of the data analysis. SM and ZX contributed to writing of the report. QL and XO contributed to critical revision of the report. LZ, YY, and HJ contributed to the statistical analysis and the data analysis portion of the manuscript. All the authors contributed to data acquisition, data analysis, or data interpretation, and reviewed and approved the final version.

## Conflict of Interest

The authors declare that the research was conducted in the absence of any commercial or financial relationships that could be construed as a potential conflict of interest.

## Footnotes

1 https://cytoscape.org/
