# Corrigendum: Identification of a Potential miRNA-mRNA Regulatory Network Associated With the Prognosis of HBV-ACLF

**DOI:** 10.3389/fmolb.2021.705683

**Published:** 2021-06-29

**Authors:** Shanshan Ma, Zhongyang Xie, Lingjian Zhang, Ya Yang, He Jiang, Xiaoxi Ouyang, Yalei Zhao, Qiuhong Liu, Xiaowei Xu, Lanjuan Li

**Affiliations:** ^1^ State Key Laboratory for Diagnosis and Treatment of Infectious Diseases, The First Affiliated Hospital, College of Medicine, Zhejiang University, Hangzhou, China; ^2^ National Clinical Research Center for Infectious Diseases, The First Affiliated Hospital, College of Medicine, Zhejiang University, Hangzhou, China; ^3^ Collaborative Innovation Center for Diagnosis and Treatment of Infectious Diseases, The First Affiliated Hospital, College of Medicine, Zhejiang University, Hangzhou, China; ^4^ Department of Infectious Diseases, The First Affiliated Hospital, College of Medicine, Zhejiang University, Hangzhou, China

**Keywords:** hepatitis B virus, acute-on-chronic liver failure, prognosis, network, miRNA

In the original article, there was an error in the **Abstract**. The second paragraph titled “**Methods**” in the **Abstract** is redundant and should have been removed. A correction has been made to the **Abstract**:

“**Background:** Hepatitis B virus-related acute-on-chronic liver failure (HBV-ACLF) is a life-threatening disease with a high mortality rate; the systemic inflammatory response plays a vital role in disease progression. We aimed to determine if a miRNA–mRNA co-regulatory network exists in the peripheral blood mononuclear cells (PBMCs) of HBV-ACLF patients, which might be important for prognosis.

**Methods:** Transcriptome-wide microRNA (miRNA) and mRNA microarrays were used to define the miRNA and mRNA expression profiles of the PBMCs of HBV-ACLF patients in a discovery cohort. The targets of the miRNAs were predicted. We built a miRNA-mRNA regulatory network through bioinformatics analysis, and used quantitative real-time polymerase chain reaction (qRT-PCR) to assess the importance of candidate miRNAs and mRNAs. We also assessed the direct and transcriptional regulatory effects of miRNAs on target mRNAs using a dual-luciferase reporter assay.

**Results:** The miRNA/mRNA PBMC expression profiles of the discovery cohort, of whom eight survived and eight died, revealed a prognostic interactive network involving 38 miRNAs and 313 mRNAs; this was constructed by identifying the target genes of the miRNAs. We validated the expression data in another cohort, of whom 43 survived and 35 died; miR-6840-3p, miR-6861-3p, JADE2, and NR3C2 were of particular interest. The levels of miR-6840-3p and miR-6861-3p were significantly increased in the PBMCs of the patients who died, and thus predicted prognosis (areas under the curve values = 0.665 and 0.700, respectively). The dual-luciferase reporter assay indicated that miR-6840-3p directly targeted JADE2.

**Conclusion:** We identified a prognostic miRNA-mRNA co-regulatory network in the PBMCs of HBV-ACLF patients. miR-6840-3p-JADE2 is a potential miRNA–mRNA pair contributing to a poor prognosis.”

The authors apologize for this error and state that this does not change the scientific conclusions of the article in any way. The original article has been updated.

