# Supplementary material for: Identification of a Potential miRNA–mRNA Regulatory Network Associated With the Prognosis of HBV-ACLF
Source: Front Mol Biosci. 2021 Apr 28;8:657631. doi: 10.3389/fmolb.2021.657631 (PMC8113841; doi:10.3389/fmolb.2021.657631)
Supplement: Supplementary file 1 [file Table_1.DOCX]

**Supplementary figures**

**Figure S1**. Differential expressions of all the 13 miRNAs of patients with HBV-ACLF in different prognosis groups of the validation cohort.

**Figure S2.** Differential expressions of all the 13 miRNAs of patients with HBV-ACLF in different grade groups of the validation cohort.

**Supplementary tables**

| **Table S1. Clinical characteristics of patients with HBV-ACLF in discovery cohort** | | | |
| --- | --- | --- | --- |
| Characteristics | Survival group (N=8) | Death group (N=8) | P Value |
| Age (years) | 36.5 (35.3, 56.0) | 63 (46.8, 67.0) | 0.038 |
| Male（No.） | 7 (87.5) | 6 (75) | 0.999 |
| BMI (kg/m^2^) | 24.8±3.5 | 24.9±4.0 | 0.975 |
| HBV-DNA level (IU/ml) | | |  |
| ＜200 | 0 | 1 | 0.073 |
| 200-2X10^4^ | 2 | 0 |  |
| 2X10^4^-2X10^6^ | 5 | 2 |  |
| ≥2X10^6^ | 1 | 5 |  |
| Laboratory data | | |  |
| WBC (10^9^/L） | 6.8±1.9 | 13±5.0 | 0.010 |
| Hb (g/L） | 125.8±19.7 | 135.8±23.5 | 0.372 |
| PLT (10^9^/L） | 115.8±53.9 | 115.5±19.6 | 0.990 |
| ALB (g/L) | 32.4+2.9 | 32.4±3.9 | 0.999 |
| ALT (U/L) | 155 (61.3, 395.0) | 330.5 (285.0, 1566.5) | 0.195 |
| AST (U/L) | 97.5 (72.3, 209.5) | 285.5 (133.3, 853.8) | 0.050 |
| TB (μmol/L) | 360±91.9 | 333.2±97.9 | 0.581 |
| Cr (μmol/L) | 58.5 (49.3, 67.8) | 78.5 (57.0, 114.3) | 0.161 |
| Na (μmol/L) | 137.1±3.4 | 137.8±5.5 | 0.790 |
| INR | 2.0±0.2 | 2.6±0.3 | 0.001 |
| AFP (μg/L) | 187.8 (122.1, 436.7) | 78.6 (13.4, 256.3) | 0.105 |
| Severity score | | |  |
| COSSH-ACLFs | 5.8±0.4 | 7.2±0.9 | 0.002 |
| CLIF-C ACLFs | 37.0±4.8 | 54.7±5.8 | ＜0.001 |
| MELD | 21.3±2.9 | 28.4±7.2 | 0.021 |
| MELD-Na | 22.0 (19.7, 24.5) | 27.6 (26.5, 33.8) | 0.005 |
| ACLF grade |  |  |  |
| 1 | 8 | 3 | 0.026 |
| 2 | 0 | 4 |  |
| 3 | 0 | 1 |  |
| Data are mean ± SD, median (p25, p75), or number (percentage). Comparisons were performed by t test, Mann−Whitney U-test, χ2 test or Fisher’s exact test. BMI, body mass index. HBV, hepatitis B virus; ACLF, acute-on-chronic liver failure; BMI, body mass index; WBC, white blood cell; Hb, hemoglobin; PLT, platelet; ALB, albumin; ALT, alanine transferase; AST, aspartate aminotransferase; TB, total bilirubin; Cr, creatinine; INR, international normalized ratio; AFP, alpha fetoprotein; COSSH, the Chinese Group on the Study of Severe Hepatitis B; MELD, model for end-stage liver disease; Na, sodium. | | | |

| **Table S2. Differentially expressed miRNAs between death group versus survival group in HBV-ACLF patients** | | | | |
| --- | --- | --- | --- | --- |
| GeneName | GeneSymbol | Regulation | log_2_FC | P Value |
| synapsin II | SYN2 | up | 2.313 | 0.000 |
| serine peptidase inhibitor, Kazal type 8 (putative) | SPINK8 | up | 2.181 | 0.000 |
| small integral membrane protein 10 | SMIM10 | up | 1.244 | 0.000 |
| peptidyl arginine deiminase, type II | PADI2 | up | 2.211 | 0.000 |
| ribonuclease, RNase A family, 2 (liver, eosinophil-derived neurotoxin) | RNASE2 | up | 1.838 | 0.000 |
| ectonucleotide pyrophosphatase/phosphodiesterase 1 | ENPP1 | down | -1.253 | 0.000 |
| lymphatic vessel endothelial hyaluronan receptor 1 | LYVE1 | up | 3.816 | 0.000 |
| peptidyl arginine deiminase, type II | PADI2 | up | 2.296 | 0.000 |
| islet cell autoantigen 1, 69kDa | ICA1 | up | 1.359 | 0.000 |
| tetratricopeptide repeat domain 24 | TTC24 | down | -1.170 | 0.000 |
| mitochondrial amidoxime reducing component 1 |  | up | 1.606 | 0.000 |
| homeodomain interacting protein kinase 2 | HIPK2 | up | 1.094 | 0.000 |
| tumor necrosis factor, alpha-induced protein 6 | TNFAIP6 | up | 2.068 | 0.000 |
| acyl-CoA oxidase 2, branched chain | ACOX2 | up | 1.380 | 0.000 |
| peroxisome proliferator-activated receptor gamma | PPARG | up | 2.269 | 0.000 |
| melanoma cell adhesion molecule | MCAM | up | 1.169 | 0.000 |
| IKAROS family zinc finger 2 (Helios) | IKZF2 | down | -1.460 | 0.000 |
| LIM homeobox 1 | LHX1 | up | 2.237 | 0.000 |
| aldehyde dehydrogenase 4 family, member A1 | ALDH4A1 | up | 1.174 | 0.000 |
| ArfGAP with FG repeats 1 | AGFG1 | up | 1.569 | 0.000 |
| solute carrier organic anion transporter family, member 2B1 | SLCO2B1 | up | 3.463 | 0.000 |
| sema domain, immunoglobulin domain (Ig), transmembrane domain (TM) and short cytoplasmic domain, (semaphorin) 4C | SEMA4C | down | -1.080 | 0.000 |
| cytochrome P450, family 19, subfamily A, polypeptide 1 | CYP19A1 | up | 3.370 | 0.000 |
| cytotoxic and regulatory T cell molecule | CRTAM | down | -1.062 | 0.000 |
| sprouty homolog 2 (Drosophila) | SPRY2 | up | 1.963 | 0.000 |
| peroxisome proliferator-activated receptor gamma | PPARG | up | 2.219 | 0.000 |
| cytidine deaminase | CDA | up | 1.029 | 0.000 |
| olfactomedin 4 | OLFM4 | up | 4.521 | 0.000 |
| GATA binding protein 3 | GATA3 | down | -1.258 | 0.000 |
| insulin | INS | up | 1.013 | 0.000 |
| leukocyte immunoglobulin-like receptor, subfamily B (with TM and ITIM domains), member 5 | LILRB5 | up | 2.663 | 0.000 |
| TIMP metallopeptidase inhibitor 4 | TIMP4 | up | 4.752 | 0.000 |
| cadherin-related family member 3 | CDHR3 | down | -1.247 | 0.000 |
| N-acetylneuraminate pyruvate lyase (dihydrodipicolinate synthase) | NPL | up | 1.123 | 0.000 |
| WD repeat domain 89 | WDR89 | down | -1.075 | 0.000 |
| arginase 1 | ARG1 | up | 3.928 | 0.000 |
| calcium activated nucleotidase 1 | CANT1 | up | 1.080 | 0.000 |
| complement component 5a receptor 2 | C5AR2 | up | 1.125 | 0.000 |
| polyhomeotic homolog 2 (Drosophila) | PHC2 | up | 1.512 | 0.000 |
| mitochondrial amidoxime reducing component 1 |  | up | 1.321 | 0.000 |
| family with sequence similarity 102, member A | FAM102A | down | -1.309 | 0.000 |
| coagulation factor XII (Hageman factor) | F12 | up | 1.086 | 0.000 |
| DEAD (Asp-Glu-Ala-Asp) box polypeptide 10 | DDX10 | down | -1.044 | 0.000 |
| spalt-like transcription factor 2 | SALL2 | down | -1.053 | 0.000 |
| polyhomeotic homolog 2 (Drosophila) | PHC2 | up | 1.682 | 0.000 |
| high mobility group box 3 | HMGB3 | up | 1.021 | 0.000 |
| signal-regulatory protein delta | SIRPD | up | 1.105 | 0.000 |
| sprouty homolog 1, antagonist of FGF signaling (Drosophila) | SPRY1 | up | 1.101 | 0.000 |
| complement component 1, q subcomponent, C chain | C1QC | up | 3.592 | 0.000 |
| jade family PHD finger 2 | JADE2 | down | -1.350 | 0.000 |
| major histocompatibility complex, class II, DQ beta 1 | HLA-DQB1 | down | -1.274 | 0.000 |
| proteinase 3 | PRTN3 | up | 3.046 | 0.000 |
| growth differentiation factor 10 | GDF10 | down | -1.577 | 0.000 |
| BMX non-receptor tyrosine kinase | BMX | up | 4.002 | 0.000 |
| MER proto-oncogene, tyrosine kinase | MERTK | up | 1.546 | 0.000 |
| zinc finger, CCHC domain containing 6 | ZCCHC6 | up | 1.127 | 0.000 |
| indoleamine 2,3-dioxygenase 2 | IDO2 | down | -1.776 | 0.000 |
| phosphatidic acid phosphatase type 2B | PPAP2B | up | 1.866 | 0.000 |
| RAS guanyl releasing protein 1 (calcium and DAG-regulated) | RASGRP1 | down | -1.038 | 0.000 |
| B-cell CLL/lymphoma 11B (zinc finger protein) | BCL11B | down | -1.378 | 0.000 |
| leucine rich repeat containing 36 | LRRC36 | down | -2.096 | 0.000 |
| leucine rich repeat neuronal 3 | LRRN3 | down | -1.976 | 0.000 |
| quiescin Q6 sulfhydryl oxidase 1 | QSOX1 | up | 1.064 | 0.000 |
| dual specificity phosphatase 13 | DUSP13 | up | 3.664 | 0.000 |
| Fc fragment of IgG binding protein | FCGBP | down | -1.916 | 0.000 |
| noggin | NOG | down | -2.217 | 0.000 |
| CD8b molecule | CD8B | down | -1.204 | 0.000 |
| prostaglandin reductase 1 | PTGR1 | up | 1.635 | 0.000 |
| human immunodeficiency virus type I enhancer binding protein 3 | HIVEP3 | down | -1.280 | 0.000 |
| sortilin-related VPS10 domain containing receptor 2 | SORCS2 | up | 1.214 | 0.000 |
| Kruppel-like factor 12 | KLF12 | down | -1.218 | 0.000 |
| RAB13, member RAS oncogene family | RAB13 | up | 1.511 | 0.000 |
| polypeptide N-acetylgalactosaminyltransferase 14 | GALNT14 | up | 2.629 | 0.000 |
| chloride intracellular channel 5 | CLIC5 | down | -1.597 | 0.000 |
| ribonuclease, RNase A family, 1 (pancreatic) | RNASE1 | up | 2.679 | 0.000 |
| CD5 molecule | CD5 | down | -1.259 | 0.000 |
| diaphanous-related formin 3 | DIAPH3 | up | 1.055 | 0.000 |
| BTB and CNC homology 1, basic leucine zipper transcription factor 2 | BACH2 | down | -1.369 | 0.000 |
| lin-7 homolog A (C. elegans) | LIN7A | up | 1.501 | 0.000 |
| growth factor receptor-bound protein 10 | GRB10 | up | 2.333 | 0.000 |
| H2.0-like homeobox | HLX | up | 1.671 | 0.000 |
| pirin (iron-binding nuclear protein) | PIR | up | 1.598 | 0.000 |
| Kruppel-like factor 12 | KLF12 | down | -1.195 | 0.000 |
| WD repeat domain 5 | WDR5 | up | 3.714 | 0.000 |
| plexin domain containing 1 | PLXDC1 | down | -1.740 | 0.000 |
| chondroitin sulfate N-acetylgalactosaminyltransferase 2 | CSGALNACT2 | up | 1.275 | 0.000 |
| NEL-like 2 (chicken) | NELL2 | down | -1.845 | 0.000 |
| NLR family, CARD domain containing 3 | NLRC3 | down | -1.288 | 0.000 |
| arginase 1 | ARG1 | up | 3.830 | 0.000 |
| CD8b molecule | CD8B | down | -1.171 | 0.000 |
| interleukin 7 receptor | IL7R | down | -1.107 | 0.000 |
| actin binding LIM protein 1 | ABLIM1 | down | -1.220 | 0.000 |
| testis specific, 10 | TSGA10 | down | -1.312 | 0.000 |
| leukotriene B4 receptor | LTB4R | up | 1.035 | 0.000 |
| calmodulin binding transcription activator 1 | CAMTA1 | down | -2.035 | 0.000 |
| cytochrome P450, family 4, subfamily F, polypeptide 2 | CYP4F2 | up | 3.110 | 0.000 |
| NDRG family member 2 | NDRG2 | down | -1.150 | 0.000 |
| IKAROS family zinc finger 2 (Helios) | IKZF2 | down | -1.506 | 0.000 |
| matrix metallopeptidase 25 | MMP25 | up | 1.978 | 0.000 |
| cholesteryl ester transfer protein, plasma | CETP | up | 1.336 | 0.000 |
| toll-like receptor 4 | TLR4 | up | 1.043 | 0.000 |
| family with sequence similarity 171, member A1 | FAM171A1 | down | -1.096 | 0.000 |
| RAB3D, member RAS oncogene family | RAB3D | up | 1.002 | 0.000 |
| CD177 molecule | CD177 | up | 5.495 | 0.000 |
| leucine rich repeat and fibronectin type III domain containing 3 | LRFN3 | down | -1.149 | 0.000 |
| CD8a molecule | CD8A | down | -1.321 | 0.000 |
| solute carrier family 38, member 10 | SLC38A10 | up | 1.043 | 0.000 |
| ST6 (alpha-N-acetyl-neuraminyl-2,3-beta-galactosyl-1,3)-N-acetylgalactosaminide alpha-2,6-sialyltransferase 3 | ST6GALNAC3 | up | 1.739 | 0.000 |
| immunoglobulin superfamily containing leucine-rich repeat 2 | ISLR2 | up | 4.364 | 0.000 |
| neuritin 1 | NRN1 | up | 2.422 | 0.000 |
| antizyme inhibitor 2 | AZIN2 | down | -1.284 | 0.000 |
| tripartite motif containing 49 | TRIM49 | down | -3.005 | 0.000 |
| pannexin 2 | PANX2 | up | 1.861 | 0.000 |
| hedgehog interacting protein | HHIP | up | 4.111 | 0.000 |
| solute carrier family 4, sodium bicarbonate transporter, member 10 | SLC4A10 | down | -2.005 | 0.000 |
| RALBP1 associated Eps domain containing 2 | REPS2 | up | 1.197 | 0.000 |
| small proline-rich protein 2F | SPRR2F | up | 2.909 | 0.000 |
| microRNA 146a | MIR146A | down | -1.391 | 0.000 |
| tumor necrosis factor receptor superfamily, member 25 | TNFRSF25 | down | -1.192 | 0.000 |
| death-associated protein kinase 2 | DAPK2 | up | 1.615 | 0.000 |
| CD2 molecule | CD2 | down | -1.199 | 0.000 |
| neuron specific gene family member 1 | NSG1 | down | -1.892 | 0.000 |
| complement component 1, r subcomponent-like | C1RL | up | 1.098 | 0.000 |
| major histocompatibility complex, class II, DQ beta 1 | HLA-DQB1 | down | -1.998 | 0.000 |
| myosin VIIA | MYO7A | up | 1.422 | 0.000 |
| transducin-like enhancer of split 3 | TLE3 | up | 1.066 | 0.000 |
| sex comb on midleg-like 4 (Drosophila) | SCML4 | down | -1.162 | 0.000 |
| matrix metallopeptidase 9 (gelatinase B, 92kDa gelatinase, 92kDa type IV collagenase) | MMP9 | up | 4.275 | 0.000 |
| CD6 molecule | CD6 | down | -1.349 | 0.000 |
| phospholipase D1, phosphatidylcholine-specific | PLD1 | up | 1.103 | 0.000 |
| tudor domain containing 9 | TDRD9 | up | 1.527 | 0.000 |
| carboxypeptidase A3 (mast cell) | CPA3 | down | -3.387 | 0.000 |
| ethanolamine kinase 2 | ETNK2 | down | -1.027 | 0.000 |
| secretory leukocyte peptidase inhibitor | SLPI | up | 1.956 | 0.000 |
| uridine phosphorylase 1 | UPP1 | up | 1.044 | 0.000 |
| cytoskeleton associated protein 2-like | CKAP2L | up | 2.504 | 0.000 |
| meiotic nuclear divisions 1 homolog (S. cerevisiae) | MND1 | up | 1.250 | 0.000 |
| amphiphysin | AMPH | up | 3.339 | 0.000 |
| RAR-related orphan receptor C | RORC | down | -1.503 | 0.000 |
| uncharacterized protein MGC40069 | MGC40069 | down | -1.467 | 0.000 |
| dachshund family transcription factor 1 | DACH1 | up | 1.848 | 0.000 |
| SH3 and PX domains 2B | SH3PXD2B | up | 1.803 | 0.000 |
| carcinoembryonic antigen-related cell adhesion molecule 4 | CEACAM4 | up | 1.385 | 0.000 |
| maltase-glucoamylase (alpha-glucosidase) | MGAM | up | 3.075 | 0.000 |
| RAR-related orphan receptor A | RORA | down | -1.204 | 0.000 |
| cell division cycle 25B | CDC25B | down | -1.044 | 0.000 |
| oleoyl-ACP hydrolase | OLAH | up | 5.198 | 0.000 |
| immunoglobin superfamily, member 21 | IGSF21 | up | 1.858 | 0.000 |
| fidgetin-like 2 | FIGNL2 | down | -1.035 | 0.000 |
| tropomyosin 2 (beta) | TPM2 | down | -1.217 | 0.000 |
| G protein-coupled receptor 160 | GPR160 | up | 1.216 | 0.000 |
| zinc finger protein 831 | ZNF831 | down | -1.006 | 0.000 |
| dachshund family transcription factor 1 | DACH1 | up | 2.048 | 0.000 |
| solute carrier family 1 (glial high affinity glutamate transporter), member 3 | SLC1A3 | up | 2.358 | 0.000 |
| Fc fragment of IgE, high affinity I, receptor for; alpha polypeptide | FCER1A | down | -4.111 | 0.000 |
| Rho GTPase activating protein 24 | ARHGAP24 | up | 1.037 | 0.000 |
| forkhead box C1 | FOXC1 | up | 1.644 | 0.000 |
| calpain 5 | CAPN5 | down | -1.261 | 0.000 |
| rootletin-like | LOC728763 | down | -1.993 | 0.000 |
| ral guanine nucleotide dissociation stimulator-like 1 | RGL1 | up | 1.613 | 0.000 |
| interleukin 1 receptor, type II | IL1R2 | up | 5.042 | 0.000 |
| solute carrier family 22, member 31 | SLC22A31 | up | 3.706 | 0.000 |
| lymphocyte antigen 9 | LY9 | down | -1.215 | 0.000 |
| ATPase, Ca++ transporting, type 2C, member 2 | ATP2C2 | up | 3.335 | 0.000 |
| high mobility group box 3 | HMGB3 | up | 1.408 | 0.000 |
| T-cell lymphoma invasion and metastasis 1 | TIAM1 | down | -1.149 | 0.000 |
| homolog of rat pragma of Rnd2 | SGK223 | down | -1.142 | 0.000 |
| limb bud and heart development | LBH | down | -1.228 | 0.000 |
| S100 calcium binding protein A12 | S100A12 | up | 1.414 | 0.000 |
| leukocyte immunoglobulin-like receptor, subfamily A (with TM domain), member 5 | LILRA5 | up | 1.119 | 0.000 |
| CD3g molecule, gamma (CD3-TCR complex) | CD3G | down | -1.383 | 0.000 |
| mast cell-expressed membrane protein 1 | MCEMP1 | up | 1.957 | 0.000 |
| CD3d molecule, delta (CD3-TCR complex) | CD3D | down | -1.174 | 0.000 |
| inhibin, beta B | INHBB | up | 2.512 | 0.000 |
| mitogen-activated protein kinase kinase 6 | MAP2K6 | up | 1.055 | 0.000 |
| proprotein convertase subtilisin/kexin type 9 | PCSK9 | up | 5.122 | 0.000 |
| chromatin licensing and DNA replication factor 1 | CDT1 | up | 1.376 | 0.000 |
| SH3 domain binding kinase 1 | SBK1 | down | -1.556 | 0.000 |
| trefoil factor 3 (intestinal) | TFF3 | up | 3.199 | 0.000 |
| coiled-coil domain containing 125 | CCDC125 | up | 1.023 | 0.000 |
| trefoil factor 3 (intestinal) | TFF3 | up | 3.212 | 0.000 |
| homeobox A10 | HOXA10 | up | 1.303 | 0.000 |
| period circadian clock 3 | PER3 | down | -1.581 | 0.000 |
| complement component 1, q subcomponent, B chain | C1QB | up | 2.618 | 0.000 |
| purinergic receptor P2Y, G-protein coupled, 1 | P2RY1 | up | 1.684 | 0.000 |
| anillin, actin binding protein | ANLN | up | 2.155 | 0.000 |
| cytochrome P450, family 4, subfamily F, polypeptide 22 | CYP4F22 | down | -1.631 | 0.000 |
| coiled-coil domain containing 17 | CCDC17 | up | 1.089 | 0.000 |
| FK506 binding protein 5 | FKBP5 | up | 1.321 | 0.000 |
| sperm associated antigen 4 | SPAG4 | up | 1.576 | 0.000 |
| syndecan 1 | SDC1 | up | 1.474 | 0.000 |
| maltase-glucoamylase (alpha-glucosidase) | MGAM | up | 2.895 | 0.000 |
| CD8b molecule | CD8B | down | -1.181 | 0.000 |
| uridine phosphorylase 1 | UPP1 | up | 1.375 | 0.000 |
| sparc/osteonectin, cwcv and kazal-like domains proteoglycan (testican) 2 | SPOCK2 | down | -1.140 | 0.000 |
| aspartate beta-hydroxylase | ASPH | up | 1.679 | 0.000 |
| tumor necrosis factor receptor superfamily, member 25 | TNFRSF25 | down | -1.170 | 0.000 |
| cytochrome P450, family 4, subfamily F, polypeptide 3 | CYP4F3 | up | 2.561 | 0.000 |
| trace amine associated receptor 5 | TAAR5 | up | 1.418 | 0.000 |
| ectonucleotide pyrophosphatase/phosphodiesterase 3 | ENPP3 | down | -2.303 | 0.000 |
| ubiquitin specific peptidase 44 | USP44 | down | -1.614 | 0.000 |
| interleukin 21 receptor | IL21R | down | -1.049 | 0.000 |
| S100 calcium binding protein A12 | S100A12 | up | 1.151 | 0.000 |
| ELOVL fatty acid elongase 4 | ELOVL4 | down | -1.615 | 0.000 |
| aspartate beta-hydroxylase | ASPH | up | 1.358 | 0.000 |
| golgin A7 family, member B | GOLGA7B | down | -1.640 | 0.001 |
| chloride intracellular channel 5 | CLIC5 | down | -1.310 | 0.001 |
| lymphoid enhancer-binding factor 1 | LEF1 | down | -1.085 | 0.001 |
| DLC1 Rho GTPase activating protein | DLC1 | up | 1.915 | 0.001 |
| tropomyosin 2 (beta) | TPM2 | down | -1.135 | 0.001 |
| chondroitin sulfate N-acetylgalactosaminyltransferase 2 | CSGALNACT2 | up | 1.108 | 0.001 |
| dysferlin | DYSF | up | 1.468 | 0.001 |
| chromosome 2 open reading frame 40 | C2orf40 | down | -1.853 | 0.001 |
| CD40 ligand | CD40LG | down | -1.775 | 0.001 |
| V-set and immunoglobulin domain containing 4 | VSIG4 | up | 3.443 | 0.001 |
| zinc finger protein 280B | ZNF280B | down | -1.099 | 0.001 |
| pyrin and HIN domain family, member 1 | PYHIN1 | down | -1.238 | 0.001 |
| Enah/Vasp-like | EVL | down | -1.152 | 0.001 |
| dihydropyrimidinase-like 3 | DPYSL3 | up | 2.661 | 0.001 |
| plexin domain containing 1 | PLXDC1 | down | -1.212 | 0.001 |
| cysteine-rich secretory protein LCCL domain containing 2 | CRISPLD2 | up | 1.297 | 0.001 |
| dpy-19-like 3 (C. elegans) | DPY19L3 | up | 1.315 | 0.001 |
| dendritic cell-associated nuclear protein | DCANP1 | down | -2.414 | 0.001 |
| WAP four-disulfide core domain 2 | WFDC2 | up | 3.158 | 0.001 |
| 5-oxoprolinase (ATP-hydrolysing) | OPLAH | up | 1.340 | 0.001 |
| solute carrier family 26 (anion exchanger), member 8 | SLC26A8 | up | 2.466 | 0.001 |
| ArfGAP with FG repeats 1 | AGFG1 | up | 1.119 | 0.001 |
| major histocompatibility complex, class II, DQ beta 2 | HLA-DQB2 | down | -1.145 | 0.001 |
| cytoskeleton-associated protein 4 | CKAP4 | up | 1.293 | 0.001 |
| annexin A3 | ANXA3 | up | 3.019 | 0.001 |
| plasminogen-like B1 | PLGLB1 | down | -1.012 | 0.001 |
| CD28 molecule | CD28 | down | -1.067 | 0.001 |
| amyloid beta (A4) precursor protein-binding, family A, member 1 | APBA1 | down | -1.880 | 0.001 |
| MANSC domain containing 1 | MANSC1 | up | 2.529 | 0.001 |
| activin A receptor type II-like 1 | ACVRL1 | up | 1.599 | 0.001 |
| interleukin 10 | IL10 | up | 2.015 | 0.001 |
| wingless-type MMTV integration site family, member 3 | WNT3 | down | -1.212 | 0.001 |
| vanin 1 | VNN1 | up | 2.225 | 0.001 |
| kinesin family member 27 | KIF27 | up | 1.068 | 0.001 |
| interleukin 5 receptor, alpha | IL5RA | down | -2.056 | 0.001 |
| KIAA0101 | KIAA0101 | up | 1.259 | 0.001 |
| gamma-glutamyl hydrolase (conjugase, folylpolygammaglutamyl hydrolase) | GGH | up | 1.634 | 0.001 |
| complement component 1, q subcomponent, A chain | C1QA | up | 1.986 | 0.001 |
| contactin associated protein-like 3 | CNTNAP3 | up | 3.145 | 0.001 |
| KIAA1211-like | KIAA1211L | up | 1.062 | 0.001 |
| contactin associated protein-like 3B | CNTNAP3B | up | 2.570 | 0.001 |
| interleukin 1 receptor, type I | IL1R1 | up | 2.095 | 0.001 |
| enoyl CoA hydratase domain containing 3 | ECHDC3 | up | 1.346 | 0.001 |
| acyl-CoA synthetase long-chain family member 1 | ACSL1 | up | 1.869 | 0.001 |
| bleomycin hydrolase | BLMH | up | 1.537 | 0.001 |
| zinc finger, DHHC-type containing 8 | ZDHHC8 | up | 2.121 | 0.001 |
| ubiquitin-like with PHD and ring finger domains 1 | UHRF1 | up | 1.156 | 0.001 |
| cadherin-related family member 3 | CDHR3 | down | -1.520 | 0.001 |
| ER lipid raft associated 1 | ERLIN1 | up | 1.163 | 0.001 |
| adrenomedullin | ADM | up | 1.979 | 0.001 |
| ADAMTS-like 3 | ADAMTSL3 | up | 4.097 | 0.001 |
| PDZ and LIM domain 7 (enigma) | PDLIM7 | up | 1.017 | 0.001 |
| Ral GTPase activating protein, alpha subunit 2 (catalytic) | RALGAPA2 | up | 1.258 | 0.001 |
| mitogen-activated protein kinase 14 | MAPK14 | up | 1.114 | 0.001 |
| epoxide hydrolase 2, cytoplasmic | EPHX2 | down | -1.248 | 0.001 |
| 5'-nucleotidase, ecto (CD73) | NT5E | down | -1.095 | 0.001 |
| leucine rich repeat (in FLII) interacting protein 1 | LRRFIP1 | up | 1.186 | 0.001 |
| carbonic anhydrase IV | CA4 | up | 4.255 | 0.001 |
| kelch-like family member 29 | KLHL29 | down | -1.052 | 0.001 |
| ZFP14 zinc finger protein | ZFP14 | down | -1.016 | 0.001 |
| small glutamine-rich tetratricopeptide repeat (TPR)-containing, alpha | SGTA | up | 1.519 | 0.001 |
| E2F transcription factor 8 | E2F8 | up | 1.913 | 0.001 |
| phosphodiesterase 7B | PDE7B | down | -1.406 | 0.001 |
| growth arrest and DNA-damage-inducible, gamma | GADD45G | up | 1.152 | 0.001 |
| ring finger protein 24 | RNF24 | up | 1.105 | 0.001 |
| nuclear receptor subfamily 3, group C, member 2 | NR3C2 | down | -1.161 | 0.001 |
| titin | TTN | down | -1.289 | 0.001 |
| cytochrome P450, family 4, subfamily F, polypeptide 8 | CYP4F8 | up | 1.387 | 0.001 |
| B-cell CLL/lymphoma 11B (zinc finger protein) | BCL11B | down | -1.133 | 0.001 |
| tumor necrosis factor (ligand) superfamily, member 11 | TNFSF11 | down | -1.731 | 0.001 |
| major histocompatibility complex, class II, DQ beta 1 | HLA-DQB1 | down | -1.205 | 0.001 |
| signal-induced proliferation-associated 1 like 2 | SIPA1L2 | up | 1.572 | 0.001 |
| growth factor receptor-bound protein 10 | GRB10 | up | 1.975 | 0.001 |
| lectin, galactoside-binding, soluble, 2 | LGALS2 | down | -2.262 | 0.001 |
| RNA binding motif protein 47 | RBM47 | up | 1.076 | 0.001 |
| solute carrier family 1 (glial high affinity glutamate transporter), member 3 | SLC1A3 | up | 2.322 | 0.001 |
| ADAM metallopeptidase with thrombospondin type 1 motif, 2 | ADAMTS2 | up | 3.979 | 0.001 |
| hematopoietic prostaglandin D synthase | HPGDS | down | -2.077 | 0.001 |
| tyrosylprotein sulfotransferase 1 | TPST1 | up | 2.409 | 0.001 |
| guanylate cyclase 1, soluble, alpha 3 | GUCY1A3 | down | -1.689 | 0.001 |
| G protein-coupled receptor 174 | GPR174 | down | -1.221 | 0.001 |
| fibulin 2 | FBLN2 | down | -1.676 | 0.001 |
| sialic acid binding Ig-like lectin 11 | SIGLEC11 | up | 1.583 | 0.001 |
| hedgehog interacting protein | HHIP | up | 3.714 | 0.001 |
| ADAM metallopeptidase with thrombospondin type 1 motif, 6 | ADAMTS6 | down | -1.196 | 0.001 |
| matrix metallopeptidase 25 | MMP25 | up | 2.226 | 0.001 |
| chitinase 1 (chitotriosidase) | CHIT1 | up | 2.804 | 0.001 |
| asialoglycoprotein receptor 2 | ASGR2 | up | 1.002 | 0.001 |
| transducin-like enhancer of split 2 | TLE2 | down | -1.283 | 0.001 |
| patatin-like phospholipase domain containing 1 | PNPLA1 | up | 1.479 | 0.001 |
| chromosome 1 open reading frame 186 | C1orf186 | down | -1.435 | 0.001 |
| TraB domain containing 2A | TRABD2A | down | -1.284 | 0.001 |
| matrix metallopeptidase 8 (neutrophil collagenase) | MMP8 | up | 4.473 | 0.001 |
| PTPN13-like, Y-linked 2 | PRY2 | down | -1.631 | 0.001 |
| branched chain amino-acid transaminase 1, cytosolic | BCAT1 | up | 1.454 | 0.001 |
| mannose receptor, C type 1 | MRC1 | up | 2.584 | 0.001 |
| signal-regulatory protein beta 1 | SIRPB1 | down | -2.204 | 0.001 |
| contactin associated protein-like 3 | CNTNAP3 | up | 2.525 | 0.001 |
| SPC25, NDC80 kinetochore complex component | SPC25 | up | 1.763 | 0.001 |
| methionine sulfoxide reductase B3 | MSRB3 | up | 1.298 | 0.001 |
| uncharacterized LOC388210 | LOC388210 | up | 1.066 | 0.001 |
| glucose-fructose oxidoreductase domain containing 1 | GFOD1 | up | 1.083 | 0.001 |
| RAD54-like (S. cerevisiae) | RAD54L | up | 1.206 | 0.001 |
| histidine decarboxylase | HDC | down | -4.613 | 0.001 |
| peptidoglycan recognition protein 1 | PGLYRP1 | up | 3.060 | 0.001 |
| checkpoint kinase 1 | CHEK1 | up | 1.067 | 0.001 |
| glutaminyl-peptide cyclotransferase | QPCT | up | 1.101 | 0.001 |
| src kinase associated phosphoprotein 1 | SKAP1 | down | -1.126 | 0.001 |
| sialic acid binding Ig-like lectin 5 | SIGLEC5 | up | 1.259 | 0.001 |
| NLR family, pyrin domain containing 9 | NLRP9 | up | 1.314 | 0.001 |
| actin binding LIM protein 1 | ABLIM1 | down | -1.070 | 0.001 |
| serpin peptidase inhibitor, clade B (ovalbumin), member 1 | SERPINB1 | up | 1.032 | 0.001 |
| branched chain amino-acid transaminase 1, cytosolic | BCAT1 | up | 1.180 | 0.001 |
| bactericidal/permeability-increasing protein | BPI | up | 2.575 | 0.001 |
| 5'-nucleotidase domain containing 2 | NT5DC2 | up | 1.293 | 0.001 |
| RAD51 recombinase | RAD51 | up | 1.107 | 0.001 |
| integral membrane protein 2A | ITM2A | down | -1.040 | 0.001 |
| LCK proto-oncogene, Src family tyrosine kinase | LCK | down | -1.077 | 0.001 |
| major histocompatibility complex, class II, DQ beta 1 | HLA-DQB1 | down | -1.019 | 0.001 |
| zinc finger protein 365 | ZNF365 | down | -1.737 | 0.001 |
| Epstein-Barr virus induced 3 | EBI3 | up | 1.687 | 0.001 |
| procollagen C-endopeptidase enhancer 2 | PCOLCE2 | up | 3.050 | 0.001 |
| P450 (cytochrome) oxidoreductase | POR | up | 1.020 | 0.001 |
| killer cell lectin-like receptor subfamily C, member 4 | KLRC4 | down | -1.347 | 0.001 |
| ADAM metallopeptidase with thrombospondin type 1 motif, 10 | ADAMTS10 | down | -1.029 | 0.001 |
| CD24 molecule | CD24 | up | 1.619 | 0.001 |
| B-cell CLL/lymphoma 6 | BCL6 | up | 1.345 | 0.001 |
| myeloperoxidase | MPO | up | 1.993 | 0.001 |
| neutrophil cytosolic factor 4, 40kDa | NCF4 | up | 1.154 | 0.001 |
| leukocyte-associated immunoglobulin-like receptor 2 | LAIR2 | down | -2.869 | 0.001 |
| nicotinamide N-methyltransferase | NNMT | up | 2.091 | 0.001 |
| glycogenin 1 | GYG1 | up | 1.381 | 0.001 |
| cell division cycle associated 5 | CDCA5 | up | 1.391 | 0.001 |
| ISL LIM homeobox 2 | ISL2 | up | 1.531 | 0.001 |
| TBC1 domain family, member 27 | TBC1D27 | up | 1.386 | 0.001 |
| glutaminyl-peptide cyclotransferase | QPCT | up | 1.187 | 0.001 |
| 5'-nucleotidase, ecto (CD73) | NT5E | down | -1.183 | 0.001 |
| elastase, neutrophil expressed | ELANE | up | 2.725 | 0.001 |
| bone morphogenetic protein 2 | BMP2 | up | 2.013 | 0.001 |
| phospholipid transfer protein | PLTP | up | 1.066 | 0.001 |
| inositol polyphosphate-4-phosphatase, type II, 105kDa | INPP4B | down | -1.247 | 0.001 |
| secreted phosphoprotein 1 | SPP1 | up | 2.574 | 0.001 |
| butyrophilin-like 8 | BTNL8 | up | 3.407 | 0.001 |
| sterile alpha motif domain containing 3 | SAMD3 | down | -1.301 | 0.001 |
| ral guanine nucleotide dissociation stimulator-like 3 | RGL3 | up | 1.531 | 0.001 |
| signal transducer and activator of transcription 4 | STAT4 | down | -1.069 | 0.001 |
| membrane-spanning 4-domains, subfamily A, member 2 | MS4A2 | down | -3.052 | 0.001 |
| centromere protein A | CENPA | up | 1.543 | 0.001 |
| cyclin-dependent kinase 1 | CDK1 | up | 1.602 | 0.001 |
| interleukin-1 receptor-associated kinase 3 | IRAK3 | up | 1.086 | 0.001 |
| diacylglycerol O-acyltransferase 2 | DGAT2 | up | 1.415 | 0.001 |
| dynein, axonemal, intermediate chain 2 | DNAI2 | down | -1.070 | 0.001 |
| WD repeat and FYVE domain containing 3 | WDFY3 | up | 1.155 | 0.001 |
| RALBP1 associated Eps domain containing 2 | REPS2 | up | 1.446 | 0.001 |
| keratin associated protein 6-3 | KRTAP6-3 | up | 2.400 | 0.001 |
| perilipin 5 | PLIN5 | up | 2.244 | 0.001 |
| TraB domain containing 2A | TRABD2A | down | -1.317 | 0.001 |
| golgin A7 family, member B | GOLGA7B | down | -1.624 | 0.001 |
| interleukin 1 receptor, type I | IL1R1 | up | 1.593 | 0.001 |
| otoferlin | OTOF | up | 1.437 | 0.001 |
| cytochrome P450, family 4, subfamily F, polypeptide 12 | CYP4F12 | up | 2.276 | 0.001 |
| N-myristoyltransferase 2 | NMT2 | down | -1.121 | 0.001 |
| R-spondin 3 | RSPO3 | up | 1.014 | 0.001 |
| klotho | KL | up | 2.125 | 0.001 |
| ankyrin repeat domain 20 family, member A11, pseudogene | ANKRD20A11P | down | -1.750 | 0.001 |
| oligodendrocyte myelin glycoprotein | OMG | up | 1.039 | 0.001 |
| germ cell associated 2 (haspin) | GSG2 | up | 1.742 | 0.001 |
| TBC1 domain family, member 4 | TBC1D4 | down | -1.149 | 0.001 |
| nucleolar protein 4-like | NOL4L | down | -1.069 | 0.001 |
| solute carrier family 45, member 3 | SLC45A3 | down | -1.572 | 0.001 |
| v-ets avian erythroblastosis virus E26 oncogene homolog | ERG | up | 2.014 | 0.001 |
| complement component 3a receptor 1 | C3AR1 | up | 1.265 | 0.001 |
| dynein regulatory complex subunit 1 | DRC1 | up | 2.173 | 0.001 |
| U6 snRNA biogenesis 1 | USB1 | up | 1.100 | 0.001 |
| denticleless E3 ubiquitin protein ligase homolog (Drosophila) | DTL | up | 1.220 | 0.001 |
| BTB and CNC homology 1, basic leucine zipper transcription factor 2 | BACH2 | down | -1.092 | 0.001 |
| spindle and kinetochore associated complex subunit 1 | SKA1 | up | 1.820 | 0.001 |
| angiomotin | AMOT | down | -1.210 | 0.001 |
| glycogenin 1 | GYG1 | up | 1.372 | 0.001 |
| transmembrane 6 superfamily member 1 | TM6SF1 | up | 1.006 | 0.001 |
| uncharacterized LOC101928448 | LOC101928448 | up | 1.501 | 0.001 |
| granulysin | GNLY | down | -1.864 | 0.001 |
| tumor-associated calcium signal transducer 2 | TACSTD2 | up | 2.378 | 0.002 |
| ring finger protein 24 | RNF24 | up | 1.054 | 0.002 |
| ADAM metallopeptidase with thrombospondin type 1 motif, 2 | ADAMTS2 | up | 2.989 | 0.002 |
| killer cell lectin-like receptor subfamily G, member 1 | KLRG1 | down | -1.668 | 0.002 |
| prostaglandin E receptor 3 (subtype EP3) | PTGER3 | down | -1.140 | 0.002 |
| pepsinogen 3, group I (pepsinogen A) | PGA3 | up | 1.221 | 0.002 |
| marker of proliferation Ki-67 | MKI67 | up | 1.362 | 0.002 |
| gelsolin | GSN | up | 1.135 | 0.002 |
| formyl peptide receptor 2 | FPR2 | up | 1.325 | 0.002 |
| CD248 molecule, endosialin | CD248 | down | -1.358 | 0.002 |
| olfactomedin-like 2B | OLFML2B | up | 1.084 | 0.002 |
| kringle containing transmembrane protein 1 | KREMEN1 | up | 1.768 | 0.002 |
| armadillo repeat containing, X-linked 4 | ARMCX4 | up | 1.227 | 0.002 |
| chondroitin sulfate N-acetylgalactosaminyltransferase 2 | CSGALNACT2 | up | 1.110 | 0.002 |
| NLR family, pyrin domain containing 6 | NLRP6 | up | 1.649 | 0.002 |
| testis specific, 10 | TSGA10 | down | -1.458 | 0.002 |
| RasGEF domain family, member 1A | RASGEF1A | up | 1.013 | 0.002 |
| metallothionein 1G | MT1G | up | 1.419 | 0.002 |
| thymocyte selection associated | THEMIS | down | -1.035 | 0.002 |
| ADAM metallopeptidase domain 9 | ADAM9 | up | 1.066 | 0.002 |
| leucine rich repeat neuronal 1 | LRRN1 | up | 2.748 | 0.002 |
| family with sequence similarity 169, member A | FAM169A | down | -1.036 | 0.002 |
| selenoprotein P, plasma, 1 | SEPP1 | up | 2.032 | 0.002 |
| carboxypeptidase E | CPE | up | 2.492 | 0.002 |
| purinergic receptor P2Y, G-protein coupled, 1 | P2RY1 | up | 1.292 | 0.002 |
| 3'-phosphoadenosine 5'-phosphosulfate synthase 2 | PAPSS2 | up | 1.131 | 0.002 |
| CDC42 effector protein (Rho GTPase binding) 1 | CDC42EP1 | down | -1.536 | 0.002 |
| murine retrovirus integration site 1 homolog | MRVI1 | up | 1.314 | 0.002 |
| tumor protein p53 inducible protein 11 | TP53I11 | up | 1.193 | 0.002 |
| grancalcin, EF-hand calcium binding protein | GCA | up | 1.306 | 0.002 |
| reticulon 4 receptor | RTN4R | down | -1.330 | 0.002 |
| high mobility group box 2 | HMGB2 | up | 1.270 | 0.002 |
| lipocalin 2 | LCN2 | up | 2.916 | 0.002 |
| laminin, gamma 1 (formerly LAMB2) | LAMC1 | up | 1.351 | 0.002 |
| perilipin 4 | PLIN4 | up | 2.758 | 0.002 |
| ring finger protein 225 | RNF225 | up | 1.308 | 0.002 |
| proline-rich transmembrane protein 4 | PRRT4 | up | 1.642 | 0.002 |
| thymidylate synthetase | TYMS | up | 1.193 | 0.002 |
| homeobox A9 | HOXA9 | up | 1.211 | 0.002 |
| neuronal cell adhesion molecule | NRCAM | down | -1.653 | 0.002 |
| transmembrane protein 106B | TMEM106B | down | -1.237 | 0.002 |
| D4, zinc and double PHD fingers family 2 | DPF2 | down | -1.107 | 0.002 |
| sterile alpha motif domain containing 3 | SAMD3 | down | -1.220 | 0.002 |
| uncharacterized LOC100128563 | LOC100128563 | up | 1.443 | 0.002 |
| SH2 domain containing 1A | SH2D1A | down | -1.164 | 0.002 |
| poliovirus receptor-related 2 (herpesvirus entry mediator B) | PVRL2 | up | 1.278 | 0.002 |
| aurora kinase A and ninein interacting protein | AUNIP | up | 1.083 | 0.002 |
| thymocyte expressed, positive selection associated 1 | TESPA1 | down | -1.015 | 0.002 |
| zinc finger protein 365 | ZNF365 | down | -1.645 | 0.002 |
| matrix metallopeptidase 19 | MMP19 | up | 1.051 | 0.002 |
| G protein-coupled receptor 97 | GPR97 | up | 2.677 | 0.002 |
| tumor protein p53 inducible protein 11 | TP53I11 | up | 1.525 | 0.002 |
| CD163 molecule-like 1 | CD163L1 | up | 1.533 | 0.002 |
| inositol(myo)-1(or 4)-monophosphatase 2 | IMPA2 | up | 1.036 | 0.002 |
| coiled-coil domain containing 102B | CCDC102B | down | -1.350 | 0.002 |
| poliovirus receptor-related 2 (herpesvirus entry mediator B) | PVRL2 | up | 1.532 | 0.002 |
| ATP-binding cassette, sub-family A (ABC1), member 13 | ABCA13 | up | 2.113 | 0.002 |
| transmembrane and immunoglobulin domain containing 3 | TMIGD3 | up | 2.905 | 0.002 |
| phosphogluconate dehydrogenase | PGD | up | 1.045 | 0.002 |
| tripartite motif containing 54 | TRIM54 | up | 1.411 | 0.002 |
| orosomucoid 2 | ORM2 | up | 1.843 | 0.002 |
| major histocompatibility complex, class II, DR alpha | HLA-DRA | down | -1.063 | 0.002 |
| arachidonate 5-lipoxygenase-activating protein | ALOX5AP | up | 1.454 | 0.002 |
| angiogenin, ribonuclease, RNase A family, 5 | ANG | up | 1.178 | 0.002 |
| leucine rich repeat containing 4 | LRRC4 | up | 1.453 | 0.002 |
| dpy-19-like 3 (C. elegans) | DPY19L3 | up | 1.124 | 0.002 |
| ankyrin repeat domain 20 family, member A2 | ANKRD20A2 | down | -1.461 | 0.002 |
| phosphodiesterase 9A | PDE9A | down | -1.256 | 0.002 |
| ribonuclease, RNase A family, 4 | RNASE4 | up | 1.150 | 0.002 |
| apoptosis-associated tyrosine kinase | AATK | up | 1.602 | 0.002 |
| WD repeat and FYVE domain containing 3 | WDFY3 | up | 1.064 | 0.002 |
| homeobox A9 | HOXA9 | up | 1.248 | 0.002 |
| cyclin J-like | CCNJL | up | 2.288 | 0.002 |
| resistin | RETN | up | 2.565 | 0.002 |
| carnosine synthase 1 | CARNS1 | down | -1.109 | 0.002 |
| WAP four-disulfide core domain 1 | WFDC1 | up | 3.287 | 0.002 |
| H1 histone family, member 0 | H1F0 | up | 1.450 | 0.002 |
| cAMP responsive element binding protein 5 | CREB5 | up | 1.104 | 0.002 |
| prostaglandin D2 receptor (DP) | PTGDR | down | -1.237 | 0.002 |
| resistin | RETN | up | 2.827 | 0.002 |
| TYMS opposite strand | TYMSOS | up | 1.656 | 0.002 |
| G protein-coupled receptor 27 | GPR27 | up | 1.569 | 0.002 |
| lactate dehydrogenase A | LDHA | up | 1.010 | 0.002 |
| UDP-GlcNAc:betaGal beta-1,3-N-acetylglucosaminyltransferase 8 | B3GNT8 | up | 1.010 | 0.002 |
| syndecan 4 | SDC4 | up | 1.602 | 0.002 |
| prolyl 3-hydroxylase 2 | P3H2 | down | -1.917 | 0.002 |
| lysophosphatidylcholine acyltransferase 2 | LPCAT2 | up | 1.171 | 0.002 |
| ADP-ribosylation factor-like 4A | ARL4A | up | 1.131 | 0.002 |
| sterile alpha motif domain containing 10 | SAMD10 | down | -1.028 | 0.002 |
| ST6 (alpha-N-acetyl-neuraminyl-2,3-beta-galactosyl-1,3)-N-acetylgalactosaminide alpha-2,6-sialyltransferase 3 | ST6GALNAC3 | up | 1.453 | 0.002 |
| calcium/calmodulin-dependent protein kinase II inhibitor 1 | CAMK2N1 | down | -1.169 | 0.003 |
| carbohydrate (N-acetylgalactosamine 4-sulfate 6-O) sulfotransferase 15 | CHST15 | up | 1.051 | 0.003 |
| RAB15, member RAS oncogene family | RAB15 | down | -1.353 | 0.003 |
| NOP2/Sun domain family, member 7 | NSUN7 | up | 2.408 | 0.003 |
| myosin VIIA | MYO7A | up | 1.850 | 0.003 |
| insulin-like growth factor binding protein 6 | IGFBP6 | down | -1.637 | 0.003 |
| T cell-interacting, activating receptor on myeloid cells 1 | TARM1 | up | 1.552 | 0.003 |
| solute carrier family 51, alpha subunit | SLC51A | up | 3.644 | 0.003 |
| SAM domain, SH3 domain and nuclear localization signals 1 | SAMSN1 | up | 1.177 | 0.003 |
| uncharacterized LOC100130078 | LOC100130078 | up | 1.395 | 0.003 |
| transmembrane protein 255A | TMEM255A | down | -2.061 | 0.003 |
| IQ motif containing GTPase activating protein 3 | IQGAP3 | up | 1.170 | 0.003 |
| tripartite motif-containing 51 | TRIM51 | down | -2.832 | 0.003 |
| solute carrier family 22 (organic anion transporter), member 7 | SLC22A7 | up | 1.303 | 0.003 |
| SH3 domain containing ring finger 3 | SH3RF3 | up | 1.031 | 0.003 |
| SPC24, NDC80 kinetochore complex component | SPC24 | up | 1.189 | 0.003 |
| solute carrier family 30 (zinc transporter), member 3 | SLC30A3 | up | 1.766 | 0.003 |
| kelch-like family member 3 | KLHL3 | down | -1.096 | 0.003 |
| hyaluronan and proteoglycan link protein 3 | HAPLN3 | down | -1.135 | 0.003 |
| peptidyl arginine deiminase, type IV | PADI4 | up | 1.561 | 0.003 |
| killer cell lectin-like receptor subfamily B, member 1 | KLRB1 | down | -1.253 | 0.003 |
| kringle containing transmembrane protein 1 | KREMEN1 | up | 2.090 | 0.003 |
| family with sequence similarity 169, member A | FAM169A | down | -1.019 | 0.003 |
| sulfotransferase family, cytosolic, 1B, member 1 | SULT1B1 | up | 1.548 | 0.003 |
| solute carrier family 16, member 14 | SLC16A14 | up | 1.761 | 0.003 |
| uncharacterized LOC101929567 | LOC101929567 | up | 1.358 | 0.003 |
| aspartate beta-hydroxylase | ASPH | up | 1.173 | 0.003 |
| growth arrest and DNA-damage-inducible, gamma | GADD45G | up | 1.217 | 0.003 |
| microtubule-associated protein 1 light chain 3 alpha | MAP1LC3A | up | 1.248 | 0.003 |
| ATPase, H+ transporting, lysosomal 42kDa, V1 subunit C1 | ATP6V1C1 | up | 1.187 | 0.003 |
| orosomucoid 1 | ORM1 | up | 1.735 | 0.003 |
| ring finger protein 144B | RNF144B | up | 1.053 | 0.003 |
| origin recognition complex, subunit 1 | ORC1 | up | 1.052 | 0.003 |
| hyaluronan-mediated motility receptor (RHAMM) | HMMR | up | 1.347 | 0.003 |
| neurexophilin 4 | NXPH4 | down | -1.510 | 0.003 |
| solute carrier family 22 (organic cation/zwitterion transporter), member 4 | SLC22A4 | up | 1.242 | 0.003 |
| prostaglandin D2 receptor 2 | PTGDR2 | down | -2.325 | 0.003 |
| formin 1 | FMN1 | up | 2.284 | 0.003 |
| collagen, type XVII, alpha 1 | COL17A1 | up | 2.668 | 0.003 |
| leucine-rich alpha-2-glycoprotein 1 | LRG1 | up | 2.007 | 0.003 |
| autophagy related 9B | ATG9B | down | -1.029 | 0.003 |
| citron rho-interacting serine/threonine kinase | CIT | up | 1.077 | 0.003 |
| GATA binding protein 2 | GATA2 | down | -2.466 | 0.003 |
| kinesin family member 3A | KIF3A | down | -1.094 | 0.003 |
| lymphotoxin beta (TNF superfamily, member 3) | LTB | down | -1.199 | 0.003 |
| CD163 molecule | CD163 | up | 1.601 | 0.003 |
| homeobox A9 | HOXA9 | up | 1.027 | 0.003 |
| BEN domain containing 7 | BEND7 | up | 1.845 | 0.003 |
| PR domain containing 5 | PRDM5 | up | 2.492 | 0.003 |
| lipoxygenase homology domains 1 | LOXHD1 | up | 1.266 | 0.003 |
| lethal giant larvae homolog 2 (Drosophila) | LLGL2 | down | -1.075 | 0.003 |
| prolyl 4-hydroxylase, alpha polypeptide II | P4HA2 | up | 1.088 | 0.003 |
| cut-like homeobox 2 | CUX2 | down | -1.604 | 0.003 |
| ankyrin repeat domain 20 family, member A1 | ANKRD20A1 | down | -1.233 | 0.003 |
| dynein, axonemal, heavy chain 14 | DNAH14 | up | 1.397 | 0.003 |
| acid sensing (proton gated) ion channel 1 | ASIC1 | down | -1.602 | 0.003 |
| germinal center-associated, signaling and motility | GCSAM | down | -1.071 | 0.003 |
| kinesin family member 14 | KIF14 | up | 1.372 | 0.003 |
| fatty acyl CoA reductase 2 | FAR2 | up | 1.304 | 0.004 |
| transcription factor 7 (T-cell specific, HMG-box) | TCF7 | down | -1.234 | 0.004 |
| PRKCQ antisense RNA 1 | PRKCQ-AS1 | down | -1.081 | 0.004 |
| A kinase (PRKA) anchor protein 12 | AKAP12 | down | -2.426 | 0.004 |
| rhophilin associated tail protein 1-like | ROPN1L | up | 1.872 | 0.004 |
| catsper channel auxiliary subunit beta | CATSPERB | down | -1.277 | 0.004 |
| carcinoembryonic antigen-related cell adhesion molecule 3 | CEACAM3 | up | 1.864 | 0.004 |
| thymidine kinase 1, soluble | TK1 | up | 1.077 | 0.004 |
| chemokine (C-X3-C motif) receptor 1 | CX3CR1 | down | -1.860 | 0.004 |
| sema domain, transmembrane domain (TM), and cytoplasmic domain, (semaphorin) 6B | SEMA6B | up | 1.523 | 0.004 |
| G protein-coupled receptor 68 | GPR68 | down | -1.273 | 0.004 |
| chemokine (C-C motif) ligand 18 (pulmonary and activation-regulated) | CCL18 | up | 2.256 | 0.004 |
| thyrotrophic embryonic factor | TEF | down | -1.068 | 0.004 |
| GRB2-related adaptor protein-like | GRAPL | down | -1.037 | 0.004 |
| kinesin family member C2 | KIFC2 | up | 1.819 | 0.004 |
| starch binding domain 1 | STBD1 | up | 1.486 | 0.004 |
| cystathionine-beta-synthase | CBS | up | 2.016 | 0.004 |
| tubulin polymerization-promoting protein family member 3 | TPPP3 | down | -1.786 | 0.004 |
| zinc finger protein 154 | ZNF154 | down | -1.116 | 0.004 |
| uncharacterized LOC100508227 | LOC100508227 | down | -1.001 | 0.004 |
| HECT, C2 and WW domain containing E3 ubiquitin protein ligase 2 | HECW2 | up | 1.135 | 0.004 |
| LIM domain kinase 2 | LIMK2 | up | 1.377 | 0.004 |
| tensin 1 | TNS1 | up | 2.351 | 0.004 |
| calcium channel, voltage-dependent, alpha 2/delta subunit 2 | CACNA2D2 | down | -1.435 | 0.004 |
| alkaline phosphatase, liver/bone/kidney | ALPL | up | 2.705 | 0.004 |
| ventricular zone expressed PH domain-containing 1 | VEPH1 | up | 1.384 | 0.004 |
| transforming growth factor, beta receptor III | TGFBR3 | down | -1.375 | 0.004 |
| flotillin 2 | FLOT2 | up | 1.162 | 0.004 |
| kinesin family member C1 | KIFC1 | up | 1.114 | 0.004 |
| wingless-type MMTV integration site family, member 1 | WNT1 | down | -1.035 | 0.004 |
| FK506 binding protein 5 | FKBP5 | up | 1.278 | 0.004 |
| transcription factor 7 (T-cell specific, HMG-box) | TCF7 | down | -1.168 | 0.004 |
| centromere protein F, 350/400kDa | CENPF | up | 1.098 | 0.004 |
| purinergic receptor P2Y, G-protein coupled, 1 | P2RY1 | up | 1.499 | 0.004 |
| fucosyltransferase 7 (alpha (1,3) fucosyltransferase) | FUT7 | up | 1.336 | 0.004 |
| cadherin-related family member 3 | CDHR3 | down | -1.508 | 0.004 |
| mitochondrial amidoxime reducing component 2 |  | up | 1.030 | 0.004 |
| DEP domain containing 1B | DEPDC1B | up | 1.347 | 0.004 |
| solute carrier family 4, sodium bicarbonate cotransporter, member 8 | SLC4A8 | down | -1.216 | 0.004 |
| adipocyte plasma membrane associated protein | APMAP | up | 1.081 | 0.004 |
| coiled-coil domain containing 102B | CCDC102B | down | -1.121 | 0.004 |
| zinc finger, DHHC-type containing 11 | ZDHHC11 | down | -1.007 | 0.004 |
| methyltransferase like 7B | METTL7B | up | 2.545 | 0.004 |
| adenylate kinase 5 | AK5 | down | -1.199 | 0.004 |
| kelch-like family member 2 | KLHL2 | up | 1.009 | 0.004 |
| formin-like 3 | FMNL3 | down | -1.288 | 0.005 |
| chemokine (C motif) ligand 1 | XCL1 | down | -1.551 | 0.005 |
| related RAS viral (r-ras) oncogene homolog 2 | RRAS2 | down | -1.011 | 0.005 |
| inscuteable homolog (Drosophila) | INSC | up | 1.905 | 0.005 |
| brain abundant, membrane attached signal protein 1 | BASP1 | up | 1.432 | 0.005 |
| calcium channel, voltage-dependent, gamma subunit 6 | CACNG6 | down | -1.990 | 0.005 |
| discs, large (Drosophila) homolog-associated protein 5 | DLGAP5 | up | 1.600 | 0.005 |
| prolactin | PRL | up | 1.941 | 0.005 |
| interleukin 18 receptor 1 | IL18R1 | up | 1.654 | 0.005 |
| carcinoembryonic antigen-related cell adhesion molecule 3 | CEACAM3 | up | 1.816 | 0.005 |
| leucine-rich repeat containing G protein-coupled receptor 6 | LGR6 | down | -1.024 | 0.005 |
| asp (abnormal spindle) homolog, microcephaly associated (Drosophila) | ASPM | up | 1.187 | 0.005 |
| growth arrest and DNA-damage-inducible, alpha | GADD45A | up | 1.621 | 0.005 |
| dishevelled associated activator of morphogenesis 2 | DAAM2 | up | 3.691 | 0.005 |
| MCF.2 cell line derived transforming sequence-like | MCF2L | down | -1.348 | 0.005 |
| proteoglycan 3 | PRG3 | up | 1.252 | 0.005 |
| cysteine-rich secretory protein 2 | CRISP2 | up | 2.213 | 0.005 |
| membrane-spanning 4-domains, subfamily A, member 2 | MS4A2 | down | -1.827 | 0.005 |
| toll-like receptor 7 | TLR7 | down | -1.087 | 0.005 |
| insulin receptor substrate 2 | IRS2 | up | 1.002 | 0.005 |
| axin 2 | AXIN2 | down | -1.275 | 0.005 |
| ADP-ribosylation factor-like 4C | ARL4C | down | -1.063 | 0.005 |
| brain expressed, X-linked 1 | BEX1 | up | 1.198 | 0.005 |
| CKLF-like MARVEL transmembrane domain containing 2 | CMTM2 | up | 1.625 | 0.005 |
| 6-phosphofructo-2-kinase/fructose-2,6-biphosphatase 3 | PFKFB3 | up | 1.203 | 0.005 |
| TCR gamma alternate reading frame protein | TARP | down | -1.021 | 0.005 |
| zinc finger, DHHC-type containing 11 | ZDHHC11 | down | -1.008 | 0.005 |
| cathepsin G | CTSG | up | 2.211 | 0.005 |
| granulysin | GNLY | down | -1.540 | 0.005 |
| thyroid hormone receptor interactor 13 | TRIP13 | up | 1.241 | 0.005 |
| G protein-coupled receptor 133 | GPR133 | down | -1.742 | 0.005 |
| chromosome 1 open reading frame 61 | C1orf61 | down | -1.225 | 0.005 |
| ubiquitin associated and SH3 domain containing A | UBASH3A | down | -1.042 | 0.005 |
| CHRNA7 (cholinergic receptor, nicotinic, alpha 7, exons 5-10) and FAM7A (family with sequence similarity 7A, exons A-E) fusion | CHRFAM7A | up | 1.095 | 0.005 |
| pyruvate dehydrogenase kinase, isozyme 4 | PDK4 | up | 1.675 | 0.005 |
| chromosome 5 open reading frame 30 | C5orf30 | up | 1.676 | 0.006 |
| BUB1 mitotic checkpoint serine/threonine kinase B | BUB1B | up | 1.228 | 0.006 |
| diaphanous-related formin 3 | DIAPH3 | up | 1.161 | 0.006 |
| transmembrane protein 236 | TMEM236 | up | 1.721 | 0.006 |
| NLR family, CARD domain containing 4 | NLRC4 | up | 1.546 | 0.006 |
| coiled-coil domain containing 102B | CCDC102B | down | -1.158 | 0.006 |
| FXYD domain containing ion transport regulator 2 | FXYD2 | down | -1.452 | 0.006 |
| toll-like receptor 3 | TLR3 | down | -1.870 | 0.006 |
| plexin domain containing 1 | PLXDC1 | down | -1.528 | 0.006 |
| transcobalamin II | TCN2 | up | 1.400 | 0.006 |
| marker of proliferation Ki-67 | MKI67 | up | 1.205 | 0.006 |
| interleukin 4 | IL4 | down | -2.410 | 0.006 |
| granzyme K (granzyme 3; tryptase II) | GZMK | down | -1.064 | 0.006 |
| cilia and flagella associated protein 58 | CFAP58 | up | 1.103 | 0.006 |
| retinoic acid receptor responder (tazarotene induced) 3 | RARRES3 | down | -1.045 | 0.006 |
| syntaxin 3 | STX3 | up | 1.301 | 0.006 |
| hepatic leukemia factor | HLF | down | -1.449 | 0.006 |
| pleckstrin homology domain containing, family F (with FYVE domain) member 1 | PLEKHF1 | down | -1.209 | 0.006 |
| A kinase (PRKA) anchor protein 12 | AKAP12 | down | -1.293 | 0.006 |
| synaptopodin | SYNPO | down | -1.196 | 0.006 |
| prune homolog 2 (Drosophila) | PRUNE2 | up | 1.737 | 0.006 |
| C-type lectin domain family 4, member E | CLEC4E | up | 1.170 | 0.006 |
| Rho guanine nucleotide exchange factor (GEF) 10-like | ARHGEF10L | down | -1.015 | 0.006 |
| protein phosphatase 2, regulatory subunit B, beta | PPP2R2B | down | -1.182 | 0.006 |
| cyclin-dependent kinase inhibitor 3 | CDKN3 | up | 1.054 | 0.006 |
| major histocompatibility complex, class II, DP beta 1 | HLA-DPB1 | down | -1.030 | 0.006 |
| kinesin family member 1B | KIF1B | up | 1.189 | 0.006 |
| pyrin and HIN domain family, member 1 | PYHIN1 | down | -1.188 | 0.006 |
| zeta-chain (TCR) associated protein kinase 70kDa | ZAP70 | down | -1.070 | 0.006 |
| uncharacterized LOC401289 | FLJ38122 | down | -1.042 | 0.006 |
| Opa interacting protein 5 | OIP5 | up | 1.075 | 0.006 |
| eomesodermin | EOMES | down | -1.195 | 0.006 |
| collagen beta(1-O)galactosyltransferase 2 | COLGALT2 | down | -1.738 | 0.006 |
| wingless-type MMTV integration site family, member 7B | WNT7B | down | -1.351 | 0.006 |
| protein C receptor, endothelial | PROCR | down | -1.090 | 0.006 |
| G-2 and S-phase expressed 1 | GTSE1 | up | 1.358 | 0.007 |
| axin 2 | AXIN2 | down | -1.178 | 0.007 |
| S100 calcium binding protein P | S100P | up | 2.343 | 0.007 |
| carcinoembryonic antigen-related cell adhesion molecule 1 (biliary glycoprotein) | CEACAM1 | up | 2.359 | 0.007 |
| phosphodiesterase 4D, cAMP-specific | PDE4D | up | 1.431 | 0.007 |
| keratin 23, type I | KRT23 | up | 1.928 | 0.007 |
| establishment of sister chromatid cohesion N-acetyltransferase 2 | ESCO2 | up | 1.507 | 0.007 |
| chromosome 1 open reading frame 226 | C1orf226 | up | 1.726 | 0.007 |
| cholinergic receptor, nicotinic, alpha 7 (neuronal) | CHRNA7 | up | 1.117 | 0.007 |
| guanine nucleotide binding protein (G protein), gamma 10 | GNG10 | up | 1.143 | 0.007 |
| aquaporin 9 | AQP9 | up | 1.148 | 0.007 |
| cytochrome P450, family 19, subfamily A, polypeptide 1 | CYP19A1 | up | 2.344 | 0.007 |
| keratin 23, type I | KRT23 | up | 1.838 | 0.007 |
| apoptosis-associated tyrosine kinase | AATK | up | 1.314 | 0.007 |
| UDP-GlcNAc:betaGal beta-1,3-N-acetylglucosaminyltransferase 5 | B3GNT5 | up | 1.395 | 0.007 |
| chemokine (C-C motif) receptor 7 | CCR7 | down | -1.027 | 0.007 |
| carcinoembryonic antigen-related cell adhesion molecule 1 (biliary glycoprotein) | CEACAM1 | up | 2.231 | 0.007 |
| male germ cell-associated kinase | MAK | up | 1.197 | 0.007 |
| serpin peptidase inhibitor, clade B (ovalbumin), member 10 | SERPINB10 | up | 2.075 | 0.007 |
| TTK protein kinase | TTK | up | 1.079 | 0.007 |
| hepatocyte growth factor (hepapoietin A; scatter factor) | HGF | up | 1.091 | 0.007 |
| SHC SH2-domain binding protein 1 | SHCBP1 | up | 1.217 | 0.007 |
| carcinoembryonic antigen-related cell adhesion molecule 7 | CEACAM7 | up | 1.504 | 0.007 |
| topoisomerase (DNA) II alpha 170kDa | TOP2A | up | 1.342 | 0.007 |
| procollagen-lysine, 2-oxoglutarate 5-dioxygenase 1 | PLOD1 | up | 1.042 | 0.007 |
| calpain 13 | CAPN13 | up | 1.061 | 0.007 |
| paired box 6 | PAX6 | up | 1.260 | 0.007 |
| sestrin 2 | SESN2 | up | 1.131 | 0.007 |
| mal, T-cell differentiation protein | MAL | down | -1.074 | 0.008 |
| nuclear receptor subfamily 4, group A, member 1 | NR4A1 | down | -2.145 | 0.008 |
| asp (abnormal spindle) homolog, microcephaly associated (Drosophila) | ASPM | up | 1.325 | 0.008 |
| iroquois homeobox 3 | IRX3 | up | 1.914 | 0.008 |
| interleukin 2 receptor, beta | IL2RB | down | -1.304 | 0.008 |
| F-box and leucine-rich repeat protein 13 | FBXL13 | up | 1.729 | 0.008 |
| deoxyribonuclease I-like 3 | DNASE1L3 | down | -1.365 | 0.008 |
| adhesion molecule with Ig-like domain 3 | AMIGO3 | up | 1.099 | 0.008 |
| bactericidal/permeability-increasing protein | BPI | up | 1.938 | 0.008 |
| transcobalamin I (vitamin B12 binding protein, R binder family) | TCN1 | up | 2.077 | 0.008 |
| polymerase (DNA directed), theta | POLQ | up | 1.045 | 0.008 |
| ADAM metallopeptidase domain 23 | ADAM23 | down | -1.599 | 0.008 |
| forkhead box M1 | FOXM1 | up | 1.215 | 0.008 |
| kinesin family member 3C | KIF3C | up | 1.226 | 0.008 |
| cell division cycle associated 2 | CDCA2 | up | 1.152 | 0.008 |
| trophoblast glycoprotein | TPBG | down | -1.495 | 0.008 |
| kinesin family member 19 | KIF19 | down | -1.758 | 0.008 |
| kin of IRRE like 3 (Drosophila) | KIRREL3 | down | -1.344 | 0.008 |
| tumor necrosis factor receptor superfamily, member 10c, decoy without an intracellular domain | TNFRSF10C | up | 1.655 | 0.008 |
| selenoprotein P, plasma, 1 | SEPP1 | up | 1.434 | 0.008 |
| ring finger protein 207 | RNF207 | down | -1.560 | 0.008 |
| fms-related tyrosine kinase 3 ligand | FLT3LG | down | -1.132 | 0.008 |
| fibronectin type III and ankyrin repeat domains 1 | FANK1 | down | -1.067 | 0.008 |
| protein kinase C and casein kinase substrate in neurons 1 | PACSIN1 | down | -1.412 | 0.008 |
| chemokine (C-X-C motif) receptor 2 | CXCR2 | up | 1.465 | 0.008 |
| dickkopf WNT signaling pathway inhibitor 3 | DKK3 | down | -1.556 | 0.008 |
| spondin 1, extracellular matrix protein | SPON1 | down | -1.445 | 0.008 |
| GLI pathogenesis-related 1 like 2 | GLIPR1L2 | down | -1.080 | 0.008 |
| cell division cycle 25A | CDC25A | up | 1.107 | 0.008 |
| arachidonate 15-lipoxygenase, type B | ALOX15B | up | 2.451 | 0.008 |
| SRY (sex determining region Y)-box 13 | SOX13 | down | -1.532 | 0.008 |
| lactotransferrin | LTF | up | 2.598 | 0.009 |
| egl-9 family hypoxia-inducible factor 3 | EGLN3 | down | -1.044 | 0.009 |
| v-ets avian erythroblastosis virus E26 oncogene homolog 2 | ETS2 | up | 1.068 | 0.009 |
| kinesin family member 15 | KIF15 | up | 1.090 | 0.009 |
| TPX2, microtubule-associated | TPX2 | up | 1.312 | 0.009 |
| ankyrin repeat domain 20 family, member A11, pseudogene | ANKRD20A11P | down | -1.482 | 0.009 |
| ubiquitin-conjugating enzyme E2C | UBE2C | up | 1.244 | 0.009 |
| small proline-rich protein 2E | SPRR2E | up | 1.443 | 0.009 |
| brain-specific angiogenesis inhibitor 2 | BAI2 | down | -1.070 | 0.009 |
| maternal embryonic leucine zipper kinase | MELK | up | 1.173 | 0.009 |
| coiled-coil and C2 domain containing 2B | CC2D2B | up | 1.020 | 0.009 |
| CD160 molecule | CD160 | down | -2.355 | 0.009 |
| PRKCQ antisense RNA 1 | PRKCQ-AS1 | down | -1.004 | 0.009 |
| interleukin 1 receptor accessory protein | IL1RAP | up | 1.452 | 0.009 |
| chromosome 1 open reading frame 159 | C1orf159 | down | -1.101 | 0.009 |
| ankyrin repeat domain 20 family, member A2 | ANKRD20A2 | down | -1.138 | 0.009 |
| thioredoxin reductase 3 | TXNRD3 | down | -1.015 | 0.009 |
| carcinoembryonic antigen-related cell adhesion molecule 6 (non-specific cross reacting antigen) | CEACAM6 | up | 2.147 | 0.009 |
| tetratricopeptide repeat domain 16 | TTC16 | down | -1.291 | 0.009 |
| proline rich 5 like | PRR5L | down | -1.252 | 0.009 |
| NLR family, pyrin domain containing 7 | NLRP7 | up | 1.377 | 0.009 |
| interleukin 1 receptor accessory protein | IL1RAP | up | 1.485 | 0.009 |
| uncharacterized LOC400999 | FLJ42351 | down | -1.041 | 0.009 |
| baculoviral IAP repeat containing 5 | BIRC5 | up | 1.296 | 0.009 |
| tripartite motif containing 32 | TRIM32 | down | -1.002 | 0.009 |
| perilipin 2 | PLIN2 | up | 1.203 | 0.009 |
| chemokine (C-X-C motif) receptor 1 | CXCR1 | up | 1.986 | 0.009 |
| carcinoembryonic antigen-related cell adhesion molecule 8 | CEACAM8 | up | 2.371 | 0.010 |
| centrosomal protein 55kDa | CEP55 | up | 1.419 | 0.010 |
| uncharacterized LOC100131581 | LOC100131581 | down | -1.262 | 0.010 |
| human immunodeficiency virus type I enhancer binding protein 3 | HIVEP3 | down | -1.102 | 0.010 |
| thioredoxin domain containing 5 (endoplasmic reticulum) | TXNDC5 | up | 2.294 | 0.010 |
| uncharacterized LOC100506191 | LOC100506191 | down | -1.030 | 0.010 |
| phospholipid scramblase 1 | PLSCR1 | up | 1.029 | 0.010 |
| zinc finger, matrin-type 4 | ZMAT4 | down | -2.078 | 0.010 |
| zinc finger protein 462 | ZNF462 | down | -1.169 | 0.010 |
| adenylate kinase 5 | AK5 | down | -1.100 | 0.010 |
| transmembrane protein 37 | TMEM37 | up | 1.095 | 0.010 |
| poliovirus receptor-related 3 | PVRL3 | down | -1.289 | 0.010 |
| kinesin family member 4A | KIF4A | up | 1.413 | 0.010 |
| cyclin-dependent kinase inhibitor 1C (p57, Kip2) | CDKN1C | down | -1.196 | 0.010 |
| stromal antigen 3 | STAG3 | up | 1.197 | 0.010 |
| free fatty acid receptor 2 | FFAR2 | up | 1.492 | 0.010 |
| transmembrane protein 132E | TMEM132E | down | -1.162 | 0.010 |
| v-myb avian myeloblastosis viral oncogene homolog-like 1 | MYBL1 | down | -1.412 | 0.010 |
| unc-45 homolog B (C. elegans) | UNC45B | up | 1.249 | 0.010 |
| KIAA1671 | KIAA1671 | down | -1.575 | 0.010 |
| killer cell lectin-like receptor subfamily G, member 1 | KLRG1 | down | -1.569 | 0.010 |
| zinc finger and BTB domain containing 46 | ZBTB46 | down | -1.130 | 0.010 |
| azurocidin 1 | AZU1 | up | 2.086 | 0.010 |
| myelin protein zero-like 2 | MPZL2 | down | -1.166 | 0.010 |
| kinesin family member 20A | KIF20A | up | 1.600 | 0.010 |
| free fatty acid receptor 3 | FFAR3 | up | 2.058 | 0.010 |
| TRAF-interacting protein with forkhead-associated domain, family member B | TIFAB | down | -2.026 | 0.010 |
| cyclin B2 | CCNB2 | up | 1.156 | 0.010 |
| phospholipase D family, member 4 | PLD4 | down | -1.342 | 0.010 |
| 6-phosphofructo-2-kinase/fructose-2,6-biphosphatase 2 | PFKFB2 | up | 2.364 | 0.010 |
| GINS complex subunit 2 (Psf2 homolog) | GINS2 | up | 1.246 | 0.010 |
| ryanodine receptor 3 | RYR3 | down | -1.047 | 0.010 |
| LY6/PLAUR domain containing 2 | LYPD2 | down | -1.954 | 0.010 |
| C-type lectin domain family 4, member D | CLEC4D | up | 1.252 | 0.011 |
| C2 calcium-dependent domain containing 2 | C2CD2 | down | -1.008 | 0.011 |
| thrombospondin 4 | THBS4 | up | 1.046 | 0.011 |
| WW domain binding protein 5 | WBP5 | up | 1.231 | 0.011 |
| zinc finger protein 257 | ZNF257 | down | -1.220 | 0.011 |
| indoleamine 2,3-dioxygenase 1 | IDO1 | down | -1.351 | 0.011 |
| mitochondrial amidoxime reducing component 2 |  | up | 1.126 | 0.011 |
| zinc finger protein 566 | ZNF566 | down | -1.146 | 0.011 |
| CD3e molecule, epsilon (CD3-TCR complex) | CD3E | down | -1.038 | 0.011 |
| transcription elongation factor A (SII), 3 | TCEA3 | down | -1.041 | 0.011 |
| kinesin family member 21A | KIF21A | down | -1.144 | 0.011 |
| calcium channel, voltage-dependent, R type, alpha 1E subunit | CACNA1E | up | 2.465 | 0.011 |
| G protein-coupled estrogen receptor 1 | GPER1 | up | 1.139 | 0.011 |
| phospholipase A2, group V | PLA2G5 | up | 1.104 | 0.011 |
| neuregulin 2 | NRG2 | down | -1.050 | 0.011 |
| transmembrane protein 236 | TMEM236 | up | 1.569 | 0.011 |
| septin 14 |  | up | 1.213 | 0.011 |
| family with sequence similarity 157, member A | FAM157A | up | 1.612 | 0.011 |
| trophinin associated protein | TROAP | up | 1.076 | 0.011 |
| putative chemokine-related protein FP248-like | LOC101930072 | down | -1.002 | 0.011 |
| peptidase inhibitor 16 | PI16 | down | -1.027 | 0.011 |
| cerebellin 3 precursor | CBLN3 | down | -1.114 | 0.012 |
| G protein-coupled receptor 20 | GPR20 | down | -1.409 | 0.012 |
| energy homeostasis associated | ENHO | down | -1.358 | 0.012 |
| marker of proliferation Ki-67 | MKI67 | up | 1.609 | 0.012 |
| G protein-coupled receptor 84 | GPR84 | up | 1.894 | 0.012 |
| apolipoprotein E | APOE | up | 1.246 | 0.012 |
| proteasome (prosome, macropain) subunit, beta type, 11 | PSMB11 | down | -1.040 | 0.012 |
| dynein, axonemal, heavy chain 14 | DNAH14 | up | 1.003 | 0.012 |
| janus kinase and microtubule interacting protein 2 | JAKMIP2 | down | -1.368 | 0.012 |
| T-box 21 | TBX21 | down | -1.336 | 0.012 |
| caveolin 1, caveolae protein, 22kDa | CAV1 | up | 1.118 | 0.012 |
| chemokine (C-C motif) ligand 5 | CCL5 | down | -1.054 | 0.012 |
| BUB1 mitotic checkpoint serine/threonine kinase | BUB1 | up | 1.274 | 0.012 |
| guanylate binding protein 4 | GBP4 | down | -1.108 | 0.012 |
| v-myb avian myeloblastosis viral oncogene homolog-like 2 | MYBL2 | up | 1.145 | 0.012 |
| AF4/FMR2 family, member 2 | AFF2 | up | 1.028 | 0.013 |
| kinesin family member 11 | KIF11 | up | 1.026 | 0.013 |
| Holliday junction recognition protein | HJURP | up | 1.212 | 0.013 |
| complement component (3b/4b) receptor 1-like | CR1L | up | 1.421 | 0.013 |
| septin 5 |  | up | 1.013 | 0.013 |
| peroxisomal biogenesis factor 11 gamma | PEX11G | up | 1.047 | 0.013 |
| zinc finger, SWIM-type containing 5 | ZSWIM5 | down | -1.122 | 0.013 |
| zinc finger protein 703 | ZNF703 | down | -1.416 | 0.013 |
| wingless-type MMTV integration site family, member 11 | WNT11 | up | 1.633 | 0.013 |
| uncharacterized LOC101928058 | LOC101928058 | down | -1.172 | 0.013 |
| G protein-coupled receptor 141 | GPR141 | up | 1.066 | 0.013 |
| polycystic kidney and hepatic disease 1 (autosomal recessive)-like 1 | PKHD1L1 | up | 1.413 | 0.013 |
| RAB40B, member RAS oncogene family | RAB40B | down | -1.038 | 0.013 |
| variable charge, X-linked 2 | VCX2 | up | 1.437 | 0.013 |
| transient receptor potential cation channel, subfamily M, member 6 | TRPM6 | up | 1.756 | 0.013 |
| antizyme inhibitor 2 | AZIN2 | down | -1.155 | 0.013 |
| spermatogenesis and centriole associated 1 | SPATC1 | up | 1.095 | 0.013 |
| tectonin beta-propeller repeat containing 2 | TECPR2 | up | 1.107 | 0.013 |
| glycerol kinase | GK | up | 1.041 | 0.014 |
| RAB3A interacting protein (rabin3)-like 1 | RAB3IL1 | up | 2.182 | 0.014 |
| solute carrier organic anion transporter family, member 4A1 | SLCO4A1 | up | 1.527 | 0.014 |
| B9 protein domain 1 | B9D1 | up | 1.230 | 0.014 |
| sterile alpha motif domain containing 12 | SAMD12 | down | -1.096 | 0.014 |
| pyrroline-5-carboxylate reductase 1 | PYCR1 | up | 1.094 | 0.014 |
| cysteine-rich transmembrane module containing 1 | CYSTM1 | up | 1.867 | 0.014 |
| transmembrane protein 244 | TMEM244 | down | -1.057 | 0.014 |
| ral guanine nucleotide dissociation stimulator-like 4 | RGL4 | up | 1.680 | 0.014 |
| solute carrier family 4 (anion exchanger), member 3 | SLC4A3 | down | -1.552 | 0.014 |
| potassium channel, inwardly rectifying subfamily J, member 15 | KCNJ15 | up | 1.716 | 0.014 |
| membrane-spanning 4-domains, subfamily A, member 4A | MS4A4A | up | 1.322 | 0.015 |
| ST8 alpha-N-acetyl-neuraminide alpha-2,8-sialyltransferase 1 | ST8SIA1 | down | -1.008 | 0.015 |
| megakaryocyte-associated tyrosine kinase | MATK | down | -1.107 | 0.015 |
| sema domain, seven thrombospondin repeats (type 1 and type 1-like), transmembrane domain (TM) and short cytoplasmic domain, (semaphorin) 5A | SEMA5A | down | -1.139 | 0.015 |
| hexokinase 3 (white cell) | HK3 | up | 1.082 | 0.015 |
| sialic acid binding Ig-like lectin 17, pseudogene | SIGLEC17P | down | -1.337 | 0.015 |
| microtubule associated tumor suppressor 1 | MTUS1 | down | -1.268 | 0.015 |
| B9 protein domain 1 | B9D1 | up | 1.162 | 0.015 |
| sphingosine-1-phosphate receptor 5 | S1PR5 | down | -1.584 | 0.015 |
| TCR gamma alternate reading frame protein | TARP | down | -1.051 | 0.016 |
| ring finger protein 207 | RNF207 | down | -1.252 | 0.016 |
| BCL2-related ovarian killer | BOK | down | -1.294 | 0.016 |
| ubiquitin carboxyl-terminal esterase L1 (ubiquitin thiolesterase) | UCHL1 | up | 2.041 | 0.016 |
| histone cluster 2, H2be | HIST2H2BE | up | 1.140 | 0.016 |
| immunoglobulin lambda-like polypeptide 5 | IGLL5 | up | 1.484 | 0.016 |
| heat shock 70kDa protein 1A | HSPA1A | up | 1.062 | 0.016 |
| interleukin 3 receptor, alpha (low affinity) | IL3RA | down | -1.115 | 0.016 |
| low density lipoprotein receptor class A domain containing 3 | LDLRAD3 | up | 1.017 | 0.016 |
| STEAP family member 1B | STEAP1B | down | -1.026 | 0.016 |
| interleukin 32 | IL32 | down | -1.094 | 0.016 |
| solute carrier family 28 (concentrative nucleoside transporter), member 3 | SLC28A3 | up | 1.689 | 0.016 |
| major histocompatibility complex, class II, DQ beta 1 | HLA-DQB1 | down | -1.193 | 0.016 |
| patched 1 | PTCH1 | down | -1.246 | 0.017 |
| chemokine (C-C motif) receptor 3 | CCR3 | down | -1.819 | 0.017 |
| cyclin A2 | CCNA2 | up | 1.202 | 0.017 |
| G protein-coupled estrogen receptor 1 | GPER1 | up | 1.367 | 0.017 |
| huntingtin interacting protein 1 | HIP1 | up | 1.283 | 0.017 |
| collagen, type XIX, alpha 1 | COL19A1 | down | -1.316 | 0.017 |
| zinc finger protein 681 | ZNF681 | down | -1.265 | 0.017 |
| vitrin | VIT | down | -1.915 | 0.017 |
| tektin 5 | TEKT5 | up | 1.515 | 0.017 |
| uncharacterized LOC388210 | LOC388210 | up | 1.120 | 0.017 |
| coiled-coil domain containing 64 | CCDC64 | down | -1.164 | 0.017 |
| glycoprotein (transmembrane) nmb | GPNMB | up | 1.061 | 0.017 |
| uncharacterized LOC102724332 | LOC102724332 | up | 1.415 | 0.017 |
| 6-phosphofructo-2-kinase/fructose-2,6-biphosphatase 3 | PFKFB3 | up | 1.177 | 0.017 |
| MAM domain containing glycosylphosphatidylinositol anchor 1 | MDGA1 | down | -2.968 | 0.017 |
| zinc finger protein 331 | ZNF331 | down | -1.006 | 0.018 |
| monoamine oxidase A | MAOA | up | 2.996 | 0.018 |
| chemokine (C-X-C motif) ligand 1 (melanoma growth stimulating activity, alpha) | CXCL1 | up | 1.626 | 0.018 |
| laminin, beta 3 | LAMB3 | up | 1.288 | 0.018 |
| SLIT-ROBO Rho GTPase activating protein 1 | SRGAP1 | up | 1.487 | 0.018 |
| killer cell lectin-like receptor subfamily C, member 3 | KLRC3 | down | -1.118 | 0.018 |
| V-set and immunoglobulin domain containing 1 | VSIG1 | down | -1.208 | 0.018 |
| centrosomal protein 55kDa | CEP55 | up | 1.341 | 0.019 |
| chemokine (C-X-C motif) ligand 1 (melanoma growth stimulating activity, alpha) | CXCL1 | up | 1.524 | 0.019 |
| dynein, cytoplasmic 2, heavy chain 1 | DYNC2H1 | down | -1.116 | 0.019 |
| carbonic anhydrase VI | CA6 | down | -1.261 | 0.019 |
| histone cluster 1, H2al | HIST1H2AL | up | 1.170 | 0.019 |
| kelch repeat and BTB (POZ) domain containing 7 | KBTBD7 | up | 1.240 | 0.019 |
| RAB17, member RAS oncogene family | RAB17 | up | 1.269 | 0.019 |
| G protein-coupled receptor 97 | GPR97 | up | 1.131 | 0.019 |
| germinal center-associated, signaling and motility-like | GCSAML | down | -1.776 | 0.019 |
| keratin 72, type II | KRT72 | down | -1.705 | 0.019 |
| tetraspanin 2 | TSPAN2 | up | 1.096 | 0.019 |
| nidogen 1 | NID1 | down | -1.255 | 0.019 |
| cathelicidin antimicrobial peptide | CAMP | up | 1.721 | 0.019 |
| StAR-related lipid transfer (START) domain containing 9 | STARD9 | down | -1.208 | 0.020 |
| interleukin 32 | IL32 | down | -1.109 | 0.020 |
| membrane bound O-acyltransferase domain containing 2 | MBOAT2 | up | 1.239 | 0.020 |
| killer cell lectin-like receptor subfamily K, member 1 | KLRK1 | down | -1.020 | 0.020 |
| multiple C2 domains, transmembrane 2 | MCTP2 | up | 1.237 | 0.020 |
| potassium channel, inwardly rectifying subfamily J, member 2 | KCNJ2 | up | 1.089 | 0.020 |
| PDZ binding kinase | PBK | up | 1.489 | 0.020 |
| Kruppel-like factor 5 (intestinal) | KLF5 | up | 1.199 | 0.020 |
| MLX interacting protein-like | MLXIPL | up | 1.155 | 0.021 |
| makorin ring finger protein 3 | MKRN3 | down | -1.517 | 0.021 |
| tetraspanin 16 | TSPAN16 | up | 1.159 | 0.021 |
| potassium channel, inwardly rectifying subfamily J, member 15 | KCNJ15 | up | 1.692 | 0.021 |
| C-type lectin domain family 4, member D | CLEC4D | up | 1.104 | 0.021 |
| G protein-coupled estrogen receptor 1 | GPER1 | up | 1.321 | 0.021 |
| pyruvate kinase, liver and RBC | PKLR | up | 1.488 | 0.021 |
| chitinase 1 (chitotriosidase) | CHIT1 | up | 1.025 | 0.022 |
| free fatty acid receptor 3 | FFAR3 | up | 1.473 | 0.022 |
| potassium channel, calcium activated intermediate/small conductance subfamily N alpha, member 3 | KCNN3 | up | 1.780 | 0.022 |
| solute carrier family 39 (zinc transporter), member 8 | SLC39A8 | up | 1.036 | 0.022 |
| milk fat globule-EGF factor 8 protein | MFGE8 | up | 1.216 | 0.022 |
| spindle and kinetochore associated complex subunit 3 | SKA3 | up | 1.806 | 0.022 |
| prokineticin 2 | PROK2 | up | 1.872 | 0.022 |
| cullin-associated and neddylation-dissociated 2 (putative) | CAND2 | down | -1.244 | 0.022 |
| lipoma HMGIC fusion partner | LHFP | up | 1.653 | 0.022 |
| 24-dehydrocholesterol reductase | DHCR24 | up | 1.008 | 0.022 |
| zinc finger protein 608 | ZNF608 | up | 1.258 | 0.022 |
| triggering receptor expressed on myeloid cells-like 2 | TREML2 | up | 1.107 | 0.022 |
| Fc fragment of IgE, high affinity I, receptor for; gamma polypeptide | FCER1G | up | 1.028 | 0.023 |
| complement component (3b/4b) receptor 1 (Knops blood group) | CR1 | up | 1.204 | 0.023 |
| leucine rich repeat and fibronectin type III domain containing 2 | LRFN2 | down | -1.024 | 0.023 |
| dysbindin (dystrobrevin binding protein 1) domain containing 1 | DBNDD1 | down | -1.478 | 0.023 |
| ankyrin repeat domain 34B | ANKRD34B | up | 1.195 | 0.023 |
| interleukin 12 receptor, beta 2 | IL12RB2 | down | -1.086 | 0.023 |
| defensin, alpha 3, neutrophil-specific | DEFA3 | up | 1.383 | 0.023 |
| potassium channel, two pore domain subfamily K, member 17 | KCNK17 | down | -1.676 | 0.023 |
| wingless-type MMTV integration site family, member 7A | WNT7A | down | -1.272 | 0.023 |
| killer cell lectin-like receptor subfamily C, member 1 | KLRC1 | down | -1.244 | 0.024 |
| epoxide hydrolase 4 | EPHX4 | down | -1.401 | 0.024 |
| Sin3A-associated protein, 30kDa | SAP30 | up | 1.387 | 0.024 |
| unc-13 homolog C (C. elegans) | UNC13C | up | 1.455 | 0.024 |
| thymosin beta 15a | TMSB15A | up | 1.168 | 0.024 |
| killer cell immunoglobulin-like receptor, two domains, short cytoplasmic tail, 4 | KIR2DS4 | up | 1.055 | 0.024 |
| contactin associated protein-like 3 | CNTNAP3 | up | 3.294 | 0.024 |
| ADAM metallopeptidase domain 12 | ADAM12 | down | -1.003 | 0.024 |
| prune homolog 2 (Drosophila) | PRUNE2 | up | 1.281 | 0.024 |
| coiled-coil domain containing 162, pseudogene | CCDC162P | up | 1.873 | 0.024 |
| janus kinase and microtubule interacting protein 2 | JAKMIP2 | down | -1.066 | 0.024 |
| desmocollin 1 | DSC1 | down | -1.224 | 0.024 |
| immunoglobulin lambda-like polypeptide 1 | IGLL1 | up | 1.504 | 0.024 |
| protein phosphatase 1, regulatory subunit 3B | PPP1R3B | up | 1.186 | 0.024 |
| interleukin 18 receptor 1 | IL18R1 | up | 1.368 | 0.024 |
| orthodenticle homeobox 1 | OTX1 | up | 1.795 | 0.025 |
| peptidase M20 domain containing 1 | PM20D1 | down | -1.136 | 0.025 |
| mannose receptor, C type 2 | MRC2 | down | -1.123 | 0.025 |
| netrin 4 | NTN4 | down | -1.279 | 0.025 |
| regulatory factor X, 2 (influences HLA class II expression) | RFX2 | up | 1.105 | 0.026 |
| urotensin 2 | UTS2 | down | -3.049 | 0.026 |
| major histocompatibility complex, class II, DP beta 2 (pseudogene) | HLA-DPB2 | down | -1.018 | 0.026 |
| sialic acid binding Ig-like lectin 12 (gene/pseudogene) | SIGLEC12 | up | 1.874 | 0.026 |
| solute carrier family 25 (mitochondrial iron transporter), member 37 | SLC25A37 | up | 1.410 | 0.026 |
| zinc finger protein 618 | ZNF618 | down | -1.012 | 0.027 |
| neurexin 2 | NRXN2 | down | -1.170 | 0.027 |
| 5-hydroxytryptamine (serotonin) receptor 1F, G protein-coupled | HTR1F | up | 1.458 | 0.027 |
| serpin peptidase inhibitor, clade I (pancpin), member 2 | SERPINI2 | down | -1.015 | 0.027 |
| kinesin family member 2C | KIF2C | up | 1.068 | 0.028 |
| thrombospondin 1 | THBS1 | up | 2.037 | 0.028 |
| Fc receptor-like 6 | FCRL6 | down | -1.407 | 0.028 |
| HEAT repeat containing 9 | HEATR9 | down | -1.183 | 0.028 |
| thrombospondin 1 | THBS1 | up | 1.934 | 0.028 |
| proline rich 29 | PRR29 | down | -1.137 | 0.028 |
| Fas ligand (TNF superfamily, member 6) | FASLG | down | -1.296 | 0.028 |
| Tctex1 domain containing 1 | TCTEX1D1 | up | 1.843 | 0.029 |
| membrane-spanning 4-domains, subfamily A, member 3 (hematopoietic cell-specific) | MS4A3 | up | 1.165 | 0.029 |
| cytidine monophosphate (UMP-CMP) kinase 2, mitochondrial | CMPK2 | down | -1.229 | 0.029 |
| Rho guanine nucleotide exchange factor (GEF) 17 | ARHGEF17 | up | 1.130 | 0.029 |
| granzyme H (cathepsin G-like 2, protein h-CCPX) | GZMH | down | -1.342 | 0.030 |
| olfactomedin 1 | OLFM1 | down | -2.254 | 0.030 |
| G protein-coupled receptor 56 | GPR56 | down | -1.399 | 0.030 |
| solute carrier family 35, member F3 | SLC35F3 | down | -1.105 | 0.030 |
| transmembrane protein 45A | TMEM45A | up | 1.885 | 0.030 |
| cadherin-like and PC-esterase domain containing 1 | CPED1 | down | -1.156 | 0.031 |
| SH2 domain containing 1B | SH2D1B | down | -1.415 | 0.031 |
| 5-oxoprolinase (ATP-hydrolysing) | OPLAH | up | 1.161 | 0.031 |
| natural cytotoxicity triggering receptor 3 | NCR3 | down | -1.261 | 0.031 |
| solute carrier family 25 (mitochondrial iron transporter), member 37 | SLC25A37 | up | 1.153 | 0.031 |
| potassium channel, two pore domain subfamily K, member 17 | KCNK17 | down | -1.575 | 0.031 |
| chromosome 8 open reading frame 46 | C8orf46 | down | -1.011 | 0.031 |
| urotensin 2 | UTS2 | down | -3.055 | 0.032 |
| neuromedin U receptor 1 | NMUR1 | down | -1.216 | 0.032 |
| insulin-like growth factor 1 (somatomedin C) | IGF1 | up | 1.717 | 0.032 |
| fms-related tyrosine kinase 3 | FLT3 | up | 1.633 | 0.032 |
| interleukin 18 receptor 1 | IL18R1 | up | 1.242 | 0.032 |
| 6-phosphofructo-2-kinase/fructose-2,6-biphosphatase 2 | PFKFB2 | up | 1.249 | 0.032 |
| HSAL5836 | LOC100130916 | down | -1.394 | 0.033 |
| protein tyrosine phosphatase, receptor type, K | PTPRK | down | -1.012 | 0.033 |
| zinc finger protein 74 | ZNF74 | down | -1.393 | 0.033 |
| NIMA-related kinase 2 | NEK2 | up | 1.230 | 0.033 |
| membrane metallo-endopeptidase | MME | up | 1.322 | 0.034 |
| chemokine (C-C motif) ligand 20 | CCL20 | up | 1.236 | 0.034 |
| adenosine A3 receptor | ADORA3 | up | 1.499 | 0.035 |
| chromosome 1 open reading frame 21 | C1orf21 | down | -1.074 | 0.035 |
| marginal zone B and B1 cell-specific protein | MZB1 | up | 1.496 | 0.035 |
| regulator of G-protein signaling 9 | RGS9 | down | -1.252 | 0.036 |
| solute carrier family 22 (organic cation/carnitine transporter), member 16 | SLC22A16 | up | 1.026 | 0.036 |
| leucine rich repeat and Ig domain containing 2 | LINGO2 | down | -1.474 | 0.036 |
| synaptopodin | SYNPO | up | 1.073 | 0.036 |
| beta-1,3-glucuronyltransferase 1 | B3GAT1 | down | -1.435 | 0.036 |
| major histocompatibility complex, class II, DQ alpha 2 | HLA-DQA2 | down | -2.040 | 0.037 |
| uncharacterized LOC647115 | FLJ36848 | up | 1.093 | 0.038 |
| transglutaminase 2 | TGM2 | up | 1.132 | 0.038 |
| transducin-like enhancer of split 1 (E(sp1) homolog, Drosophila) | TLE1 | up | 1.065 | 0.039 |
| interleukin 18 receptor accessory protein | IL18RAP | up | 1.366 | 0.039 |
| serpin peptidase inhibitor, clade B (ovalbumin), member 2 | SERPINB2 | up | 1.533 | 0.039 |
| LIM domain kinase 2 | LIMK2 | up | 1.004 | 0.039 |
| complement component (3d/Epstein Barr virus) receptor 2 | CR2 | down | -1.029 | 0.040 |
| WAP, follistatin/kazal, immunoglobulin, kunitz and netrin domain containing 1 | WFIKKN1 | up | 1.237 | 0.040 |
| mitochondrial calcium uptake family, member 3 | MICU3 | down | -1.083 | 0.040 |
| v-myb avian myeloblastosis viral oncogene homolog-like 1 | MYBL1 | down | -1.169 | 0.041 |
| hippocalcin like 4 | HPCAL4 | down | -1.088 | 0.041 |
| epithelial membrane protein 2 | EMP2 | up | 1.100 | 0.041 |
| MAX dimerization protein 3 | MXD3 | up | 1.244 | 0.041 |
| coiled-coil and C2 domain containing 2A | CC2D2A | down | -1.036 | 0.041 |
| dynein, axonemal, heavy chain 10 | DNAH10 | up | 1.367 | 0.041 |
| thioredoxin domain containing 5 (endoplasmic reticulum) | TXNDC5 | up | 1.080 | 0.041 |
| histone cluster 2, H3a | HIST2H3A | up | 1.301 | 0.042 |
| killer cell lectin-like receptor subfamily F, member 1 | KLRF1 | down | -1.269 | 0.042 |
| olfactomedin 1 | OLFM1 | down | -2.199 | 0.043 |
| Fc receptor-like 6 | FCRL6 | down | -1.334 | 0.045 |
| plexin B3 | PLXNB3 | up | 1.299 | 0.045 |
| protease, serine, 23 | PRSS23 | down | -1.122 | 0.046 |
| defensin, alpha 4, corticostatin | DEFA4 | up | 1.552 | 0.046 |
| immunoglobulin J polypeptide, linker protein for immunoglobulin alpha and mu polypeptides | IGJ | up | 1.458 | 0.046 |
| kelch-like family member 30 | KLHL30 | down | -1.251 | 0.047 |
| phospholipase C, gamma 1 | PLCG1 | down | -1.067 | 0.047 |
| amphiregulin | AREG | up | 1.693 | 0.048 |
| epiregulin | EREG | up | 1.552 | 0.049 |
| interleukin 18 receptor accessory protein | IL18RAP | up | 1.204 | 0.049 |
| phosphate regulating endopeptidase homolog, X-linked | PHEX | down | -1.039 | 0.049 |
| transient receptor potential cation channel, subfamily M, member 6 | TRPM6 | up | 1.566 | 0.049 |
| killer cell lectin-like receptor subfamily D, member 1 | KLRD1 | down | -1.094 | 0.050 |
| fibroblast growth factor 13 | FGF13 | up | 2.053 | 0.050 |
| HBV, hepatitis B virus; ACLF, acute-on-chronic liver failure. | | | | |

| **Table S3. Interaction of miRNAs and mRNAs between death cases and survival controls in patients with HBV-ACLF** | | | | | |
| --- | --- | --- | --- | --- | --- |
| miRNA_ID | miRNA_  Regulation | miRNA_log_2_FC | P Value | Number of target mRNA | Target mRNA_Regulation |
| hsa-let-7f-1-3p | Up | 1.682 | 0.034 | 3 | Down |
| hsa-miR-1246 | Up | 2.134 | 0.002 | 6 | Down |
| hsa-miR-1260a | Up | 1.038 | 0.012 | 8 | Down |
| hsa-miR-199b-5p | Up | 1.361 | 0.014 | 7 | Down |
| hsa-miR-223-5p | Up | 1.229 | 0.030 | 5 | Down |
| hsa-miR-3151-3p | Up | -1.148 | 0.005 | 3 | Down |
| hsa-miR-3190-5p | Up | 1.707 | 0.041 | 13 | Down |
| hsa-miR-33b-3p | Up | 1.471 | 0.041 | 15 | Down |
| hsa-miR-3663-3p | Up | 3.356 | 0.026 | 9 | Down |
| hsa-miR-424-5p | Up | 1.855 | 0.037 | 16 | Down |
| hsa-miR-4436b-5p | Up | 2.773 | 0.001 | 13 | Down |
| hsa-miR-4484 | Up | 2.733 | 0.007 | 4 | Down |
| hsa-miR-4516 | Up | 1.844 | 0.003 | 4 | Down |
| hsa-miR-4666b | Up | 2.291 | 0.018 | 5 | Down |
| hsa-miR-4698 | Up | 2.121 | 0.027 | 13 | Down |
| hsa-miR-4750-3p | Up | 1.421 | 0.048 | 3 | Down |
| hsa-miR-5010-3p | Up | 2.798 | 0.001 | 1 | Down |
| hsa-miR-563 | Up | 1.526 | 0.046 | 1 | Down |
| hsa-miR-6085 | Up | 1.127 | 0.000 | 28 | Down |
| hsa-miR-6126 | Up | 2.227 | 0.000 | 2 | Down |
| hsa-miR-634 | Up | 1.244 | 0.018 | 8 | Down |
| hsa-miR-642a-3p | Up | 3.273 | 0.010 | 9 | Down |
| hsa-miR-664a-3p | Up | 2.936 | 0.000 | 10 | Down |
| hsa-miR-6785-3p | Up | 1.662 | 0.041 | 2 | Down |
| hsa-miR-6840-3p | Up | 3.002 | 0.019 | 20 | Down |
| hsa-miR-6861-3p | Up | 2.369 | 0.004 | 4 | Down |
| hsa-miR-98-3p | Up | 1.504 | 0.037 | 7 | Down |
| hsa-miR-1268a | Down | -2.445 | 0.004 | 13 | Up |
| hsa-miR-1275 | Down | -2.297 | 0.001 | 4 | Up |
| hsa-miR-210-3p | Down | -2.261 | 0.004 | 6 | Up |
| hsa-miR-29b-1-5p | Down | -1.320 | 0.017 | 20 | Up |
| hsa-miR-342-3p | Down | -1.032 | 0.002 | 5 | Up |
| hsa-miR-342-5p | Down | -1.803 | 0.016 | 5 | Up |
| hsa-miR-361-3p | Down | -1.513 | 0.048 | 30 | Up |
| hsa-miR-3656 | Down | -2.906 | 0.004 | 6 | Up |
| hsa-miR-4323 | Down | -1.130 | 0.002 | 5 | Up |
| hsa-miR-4739 | Down | -2.193 | 0.006 | 95 | Up |
| hsa-miR-98-5p | Down | -2.062 | 0.008 | 9 | Up |

| **Table S4. Correlation analysis of prognosis related miRNA mRNA network in PBMCs of patients with HBV-ACLF** | | | | | |
| --- | --- | --- | --- | --- | --- |
| Gene symbol | log_2_FC | p value | Corresponding miRNA | Correlation coefficient | P value for correlation |
| ZDHHC8 | 2.121 | 6.629E-04 | miR-342-3p | -0.867 | ＜0.001 |
| METTL7B | 2.545 | 4.458E-03 | miR-4323 | -0.861 | ＜0.001 |
| RNASE1 | 2.679 | 1.115E-04 | miR-4739 | -0.846 | ＜0.001 |
| SLC22A7 | 1.303 | 2.652E-03 | miR-342-3p | -0.806 | ＜0.001 |
| PVRL2 | 1.278 | 1.975E-03 | miR-342-3p | -0.799 | ＜0.001 |
| AMPH | 3.339 | 2.632E-04 | miR-4739 | -0.769 | ＜0.001 |
| NNMT | 2.091 | 1.133E-03 | miR-361-3p | -0.767 | 0.001 |
| IKZF2 | -1.460 | 1.550E-05 | miR-424-5p | -0.767 | 0.001 |
| DCANP1 | -2.414 | 5.576E-04 | miR-4436b-5p | -0.754 | 0.001 |
| PRRT4 | 1.642 | 1.903E-03 | miR-4739 | -0.752 | 0.001 |
| FCGBP | -1.916 | 9.234E-05 | miR-6085 | -0.749 | 0.001 |
| RORA | -1.204 | 2.788E-04 | miR-6126 | -0.739 | 0.001 |
| COL17A1 | 2.668 | 3.081E-03 | miR-3151-3p | -0.726 | 0.001 |
| MMP19 | 1.051 | 2.057E-03 | miR-4739 | -0.722 | 0.002 |
| BACH2 | -1.369 | 1.154E-04 | miR-223-5p | -0.721 | 0.002 |
| GCSAM | -1.071 | 3.469E-03 | miR-5010-3p | -0.714 | 0.002 |
| CDC25B | -1.044 | 2.836E-04 | miR-4436b-5p | -0.705 | 0.002 |
| CHEK1 | 1.067 | 9.834E-04 | miR-342-3p | -0.696 | 0.003 |
| FBLN2 | -1.676 | 8.566E-04 | miR-4436b-5p | -0.696 | 0.003 |
| C2orf40 | -1.853 | 5.321E-04 | miR-4436b-5p | -0.691 | 0.003 |
| SEMA6B | 1.523 | 3.734E-03 | miR-1268a | -0.690 | 0.003 |
| THBS4 | 1.046 | 1.057E-02 | miR-1268a | -0.690 | 0.003 |
| ACVRL1 | 1.599 | 6.177E-04 | miR-3151-3p | -0.689 | 0.003 |
| HIVEP3 | -1.280 | 1.015E-04 | miR-4516 | -0.689 | 0.003 |
| FBLN2 | -1.676 | 8.566E-04 | miR-6861-3p | -0.689 | 0.003 |
| FAM171A1 | -1.096 | 1.786E-04 | miR-6085 | -0.688 | 0.003 |
| BCAT1 | 1.454 | 9.355E-04 | miR-98-5p | -0.687 | 0.003 |
| GPR56 | -1.399 | 2.970E-02 | miR-424-5p | -0.683 | 0.004 |
| SIGLEC11 | 1.583 | 8.665E-04 | miR-4739 | -0.681 | 0.004 |
| JADE2 | -1.350 | 6.031E-05 | miR-424-5p | -0.675 | 0.004 |
| CD8A | -1.321 | 1.859E-04 | miR-6085 | -0.671 | 0.004 |
| ARHGEF10L | -1.015 | 6.084E-03 | miR-199b-5p | -0.668 | 0.005 |
| TRIM32 | -1.002 | 9.470E-03 | miR-4436b-5p | -0.666 | 0.005 |
| ZDHHC11 | -1.007 | 4.458E-03 | miR-6085 | -0.664 | 0.005 |
| ASPH | 1.679 | 4.480E-04 | miR-361-3p | -0.661 | 0.005 |
| CDC42EP1 | -1.536 | 1.772E-03 | miR-6085 | -0.659 | 0.006 |
| TESPA1 | -1.015 | 2.009E-03 | miR-199b-5p | -0.657 | 0.006 |
| RYR3 | -1.047 | 1.048E-02 | miR-199b-5p | -0.655 | 0.006 |
| NLRC3 | -1.288 | 1.339E-04 | miR-199b-5p | -0.653 | 0.006 |
| HIVEP3 | -1.280 | 1.015E-04 | miR-6085 | -0.650 | 0.006 |
| NLRP6 | 1.649 | 1.609E-03 | miR-210-3p | -0.646 | 0.007 |
| TTC24 | -1.170 | 7.249E-06 | miR-1260a | -0.645 | 0.007 |
| NSG1 | -1.892 | 2.343E-04 | miR-4436b-5p | -0.645 | 0.007 |
| TTN | -1.289 | 7.585E-04 | miR-223-5p | -0.644 | 0.007 |
| FMNL3 | -1.288 | 4.520E-03 | miR-424-5p | -0.644 | 0.007 |
| PLXDC1 | -1.740 | 1.283E-04 | miR-6085 | -0.643 | 0.007 |
| KLHL29 | -1.052 | 7.072E-04 | miR-6085 | -0.640 | 0.008 |
| FAM169A | -1.036 | 1.682E-03 | miR-4436b-5p | -0.639 | 0.008 |
| CD3G | -1.383 | 3.516E-04 | miR-6085 | -0.637 | 0.008 |
| DPYSL3 | 2.661 | 5.423E-04 | miR-342-5p | -0.635 | 0.008 |
| SIGLEC12 | 1.874 | 2.603E-02 | miR-4739 | -0.634 | 0.008 |
| RGL1 | 1.613 | 3.199E-04 | miR-361-3p | -0.632 | 0.009 |
| MICU3 | -1.083 | 3.997E-02 | miR-4516 | -0.632 | 0.009 |
| NLRP7 | 1.377 | 9.307E-03 | miR-342-3p | -0.630 | 0.009 |
| NR3C2 | -1.161 | 7.564E-04 | miR-6085 | -0.629 | 0.009 |
| ADAMTS2 | 3.979 | 8.085E-04 | miR-4739 | -0.628 | 0.009 |
| SLC4A10 | -2.005 | 2.176E-04 | miR-4484 | -0.624 | 0.010 |
| KLHL3 | -1.096 | 2.753E-03 | miR-424-5p | -0.622 | 0.010 |
| PDE7B | -1.406 | 7.321E-04 | miR-199b-5p | -0.621 | 0.010 |
| ZNF566 | -1.146 | 1.077E-02 | miR-4516 | -0.620 | 0.010 |
| TCN2 | 1.400 | 5.769E-03 | miR-4739 | -0.618 | 0.011 |
| RNF225 | 1.308 | 1.897E-03 | miR-4323 | -0.607 | 0.013 |
| NR3C2 | -1.161 | 7.564E-04 | miR-223-5p | -0.604 | 0.013 |
| BCL6 | 1.345 | 1.102E-03 | miR-361-3p | -0.604 | 0.013 |
| SORCS2 | 1.214 | 1.040E-04 | miR-4739 | -0.604 | 0.013 |
| GOLGA7B | -1.640 | 5.016E-04 | miR-6085 | -0.601 | 0.014 |
| SEMA6B | 1.523 | 3.734E-03 | miR-4739 | -0.600 | 0.014 |
| ERG | 2.014 | 1.379E-03 | miR-1268a | -0.598 | 0.014 |
| MYO7A | 1.422 | 2.406E-04 | miR-361-3p | -0.598 | 0.014 |
| CAPN5 | -1.261 | 3.093E-04 | miR-3663-3p | -0.597 | 0.015 |
| FBLN2 | -1.676 | 8.566E-04 | miR-33b-3p | -0.596 | 0.015 |
| CAPN5 | -1.261 | 3.093E-04 | miR-6085 | -0.596 | 0.015 |
| ICA1 | 1.359 | 7.057E-06 | miR-361-3p | -0.595 | 0.015 |
| SLC1A3 | 2.358 | 3.050E-04 | miR-4739 | -0.595 | 0.015 |
| SLC1A3 | 2.358 | 3.050E-04 | miR-4739 | -0.595 | 0.015 |
| SPOCK2 | -1.140 | 4.441E-04 | miR-6085 | -0.595 | 0.015 |
| KIAA1211L | 1.062 | 6.358E-04 | miR-4739 | -0.593 | 0.015 |
| JAKMIP2 | -1.368 | 1.192E-02 | miR-4436b-5p | -0.591 | 0.016 |
| LRG1 | 2.007 | 3.089E-03 | miR-361-3p | -0.589 | 0.016 |
| MTUS1 | -1.268 | 1.543E-02 | miR-4436b-5p | -0.587 | 0.017 |
| GDF10 | -1.577 | 6.224E-05 | miR-6085 | -0.586 | 0.017 |
| CACNA2D2 | -1.435 | 4.010E-03 | miR-3663-3p | -0.579 | 0.019 |
| GCSAM | -1.071 | 3.469E-03 | miR-6085 | -0.578 | 0.019 |
| SORCS2 | 1.214 | 1.040E-04 | miR-29b-1-5p | -0.574 | 0.020 |
| CD28 | -1.067 | 6.082E-04 | miR-634 | -0.574 | 0.020 |
| ECHDC3 | 1.346 | 6.538E-04 | miR-98-5p | -0.574 | 0.020 |
| C1RL | 1.098 | 2.350E-04 | miR-361-3p | -0.573 | 0.020 |
| TBC1D4 | -1.149 | 1.365E-03 | miR-6861-3p | -0.568 | 0.022 |
| SIGLEC11 | 1.583 | 8.665E-04 | miR-29b-1-5p | -0.567 | 0.022 |
| SDC4 | 1.602 | 2.428E-03 | miR-4739 | -0.567 | 0.022 |
| NR3C2 | -1.161 | 7.564E-04 | miR-6861-3p | -0.566 | 0.022 |
| SLC38A10 | 1.043 | 1.914E-04 | miR-361-3p | -0.565 | 0.023 |
| PBK | 1.489 | 2.025E-02 | miR-361-3p | -0.565 | 0.023 |
| RAB15 | -1.353 | 2.521E-03 | miR-3190-5p | -0.564 | 0.023 |
| QSOX1 | 1.064 | 9.130E-05 | miR-1275 | -0.562 | 0.023 |
| HDC | -4.613 | 9.743E-04 | miR-4484 | -0.561 | 0.024 |
| OPLAH | 1.340 | 5.592E-04 | miR-361-3p | -0.559 | 0.024 |
| HOXA9 | 1.211 | 1.909E-03 | miR-4739 | -0.559 | 0.024 |
| CLEC4D | 1.252 | 1.051E-02 | miR-361-3p | -0.557 | 0.025 |
| LRRC36 | -2.096 | 7.654E-05 | miR-1246 | -0.554 | 0.026 |
| B9D1 | 1.230 | 1.374E-02 | miR-1275 | -0.554 | 0.026 |
| SERPINB1 | 1.032 | 1.008E-03 | miR-342-5p | -0.553 | 0.026 |
| CAND2 | -1.244 | 2.218E-02 | miR-33b-3p | -0.550 | 0.027 |
| HHIP | 4.111 | 2.088E-04 | miR-4739 | -0.547 | 0.028 |
| CDT1 | 1.376 | 3.801E-04 | miR-361-3p | -0.542 | 0.030 |
| TP53I11 | 1.193 | 1.807E-03 | miR-3151-3p | -0.540 | 0.031 |
| TTN | -1.289 | 7.585E-04 | miR-424-5p | -0.538 | 0.032 |
| ISLR2 | 4.364 | 1.971E-04 | miR-4739 | -0.538 | 0.031 |
| SYN2 | 2.313 | 4.824E-07 | miR-29b-1-5p | -0.536 | 0.032 |
| C1orf21 | -1.074 | 3.493E-02 | miR-223-5p | -0.535 | 0.033 |
| SLC30A3 | 1.766 | 2.741E-03 | miR-4739 | -0.533 | 0.034 |
| TTN | -1.289 | 7.585E-04 | miR-6840-3p | -0.533 | 0.034 |
| ZDHHC8 | 2.121 | 6.629E-04 | miR-4739 | -0.532 | 0.034 |
| FCGBP | -1.916 | 9.234E-05 | miR-3190-5p | -0.531 | 0.034 |
| DPYSL3 | 2.661 | 5.423E-04 | miR-4739 | -0.531 | 0.034 |
| ENPP1 | -1.253 | 5.133E-06 | miR-98-3p | -0.531 | 0.034 |
| C1QC | 3.592 | 5.966E-05 | miR-4739 | -0.530 | 0.035 |
| THBS1 | 2.037 | 2.777E-02 | miR-4739 | -0.530 | 0.035 |
| SBK1 | -1.556 | 3.808E-04 | miR-6085 | -0.527 | 0.036 |
| ZFP14 | -1.016 | 7.190E-04 | miR-664a-3p | -0.527 | 0.036 |
| MRC2 | -1.123 | 2.516E-02 | miR-6840-3p | -0.527 | 0.036 |
| KIF3C | 1.226 | 7.816E-03 | miR-361-3p | -0.526 | 0.036 |
| GPR97 | 2.677 | 2.059E-03 | miR-361-3p | -0.525 | 0.037 |
| NRN1 | 2.422 | 2.000E-04 | miR-4739 | -0.525 | 0.037 |
| AGFG1 | 1.569 | 1.988E-05 | miR-4739 | -0.525 | 0.037 |
| KLHL3 | -1.096 | 2.753E-03 | miR-6085 | -0.525 | 0.037 |
| CRISP2 | 2.213 | 5.068E-03 | miR-1275 | -0.523 | 0.038 |
| JADE2 | -1.350 | 6.031E-05 | miR-6840-3p | -0.522 | 0.038 |
| TTN | -1.289 | 7.585E-04 | miR-4436b-5p | -0.520 | 0.039 |
| NELL2 | -1.845 | 1.298E-04 | miR-4698 | -0.520 | 0.039 |
| AXIN2 | -1.275 | 5.187E-03 | miR-424-5p | -0.519 | 0.040 |
| OTX1 | 1.795 | 2.451E-02 | miR-4739 | -0.518 | 0.040 |
| C1orf226 | 1.726 | 6.637E-03 | miR-4739 | -0.517 | 0.040 |
| BCL11B | -1.378 | 7.413E-05 | miR-6840-3p | -0.516 | 0.041 |
| DAAM2 | 3.691 | 4.992E-03 | miR-361-3p | -0.515 | 0.041 |
| TACSTD2 | 2.378 | 1.501E-03 | miR-4739 | -0.515 | 0.041 |
| CEACAM6 | 2.147 | 9.253E-03 | miR-4739 | -0.514 | 0.042 |
| SDC1 | 1.474 | 4.359E-04 | miR-4323 | -0.512 | 0.043 |
| BLMH | 1.537 | 6.602E-04 | miR-4739 | -0.511 | 0.043 |
| TYMSOS | 1.656 | 2.382E-03 | miR-4739 | -0.510 | 0.044 |
| ABCA13 | 2.113 | 2.117E-03 | miR-4739 | -0.507 | 0.045 |
| ABLIM1 | -1.220 | 1.485E-04 | miR-6840-3p | -0.507 | 0.045 |
| GSG2 | 1.742 | 1.362E-03 | miR-361-3p | -0.505 | 0.046 |
| TRABD2A | -1.284 | 9.288E-04 | miR-634 | -0.505 | 0.046 |
| PDLIM7 | 1.017 | 6.859E-04 | miR-361-3p | -0.503 | 0.047 |
| KRT72 | -1.705 | 1.924E-02 | miR-424-5p | -0.503 | 0.047 |
| TXNRD3 | -1.015 | 9.213E-03 | miR-6785-3p | -0.502 | 0.047 |
| CDCA2 | 1.152 | 7.838E-03 | miR-342-5p | -0.501 | 0.048 |
| E2F8 | 1.913 | 7.279E-04 | miR-361-3p | -0.501 | 0.048 |
| PTGDR | -1.237 | 2.351E-03 | miR-3663-3p | -0.501 | 0.048 |
| GPR68 | -1.273 | 3.756E-03 | miR-634 | -0.499 | 0.049 |
| DCANP1 | -2.414 | 5.576E-04 | miR-3190-5p | -0.498 | 0.050 |
| SH2D1B | -1.415 | 3.077E-02 | miR-424-5p | -0.497 | 0.050 |
