# Supplementary figures and images for: Identification of a Potential miRNA–mRNA Regulatory Network Associated With the Prognosis of HBV-ACLF
Source: Front Mol Biosci. 2021 Apr 28;8:657631. doi: 10.3389/fmolb.2021.657631 (PMC8113841; doi:10.3389/fmolb.2021.657631)

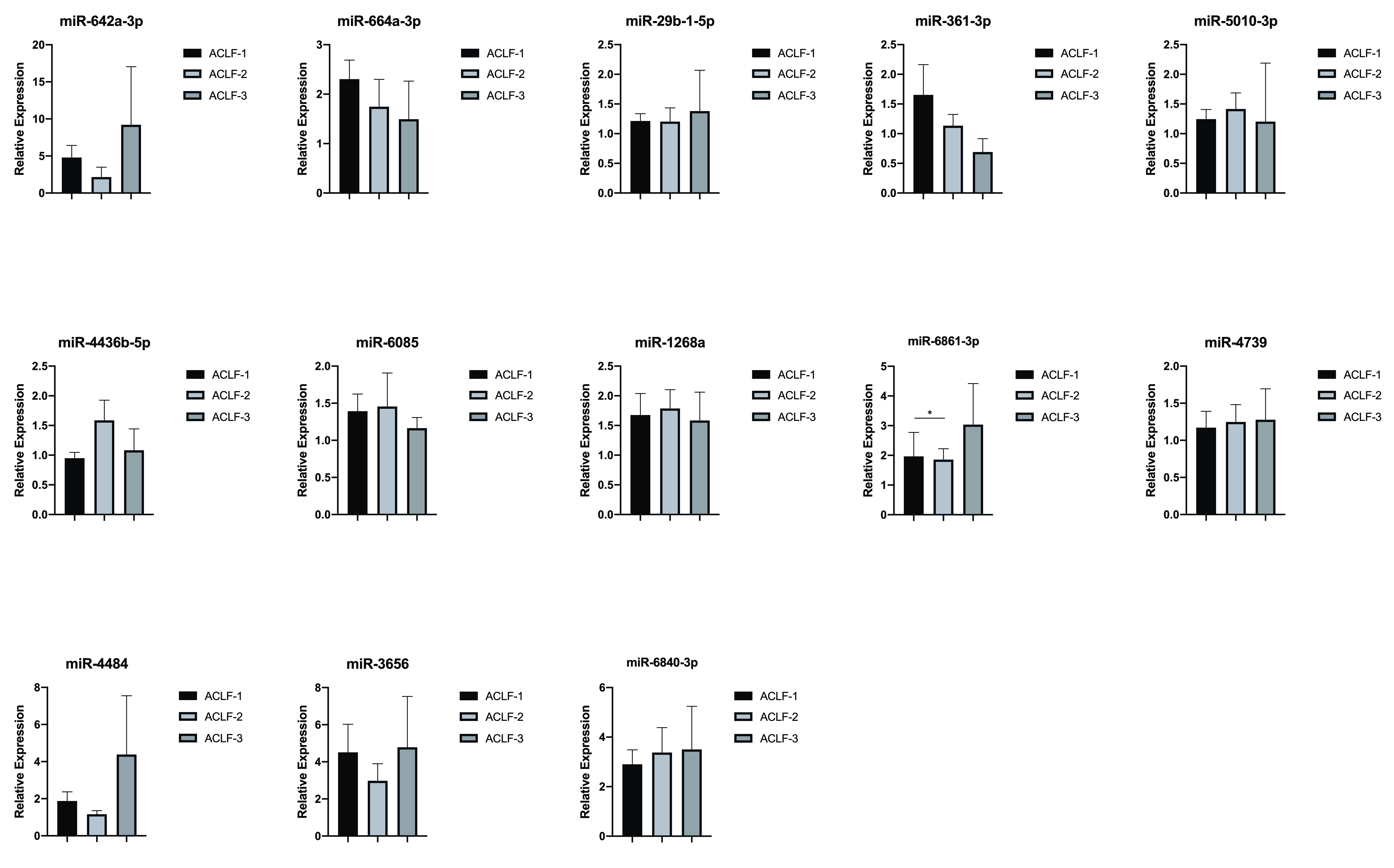

Supplement: Supplementary file 3 [file Image_2.TIFF]
